# METTL5‐mediated 18S rRNA m^6^A modification enhances ribosome assembly and ABA response in *Arabidopsis*

**DOI:** 10.1002/imt2.70055

**Published:** 2025-06-13

**Authors:** Ping Li, Yu Zhang, Songyao Zhang, Jinqi Ma, Sheng Fan, Lisha Shen

**Affiliations:** ^1^ Temasek Life Sciences Laboratory National University of Singapore Singapore Singapore; ^2^ Department of Biological Sciences National University of Singapore Singapore Singapore

## Abstract

METTL5 catalyzes the *N* ^6^‐methyladenosine (m^6^A) methylation at A_1771_ in 18S rRNA, a modification essential for its association with the ribosomal protein RPL24A, facilitating the assembly of 80S ribosome. This facilitates the translation of mRNAs encoding the detoxifying glutathione S‐transferase (GST) enzymes, thereby maintaining normal reactive oxygen species (ROS) levels and ensuring proper abscisic acid (ABA) responses. In *mettl5* mutants, the absence of m^6^A_1771_ compromises RPL24A incorporation and ribosome assembly, impairing the translation of GSTs. This results in ROS excessive accumulation and hypersensitivity to ABA.

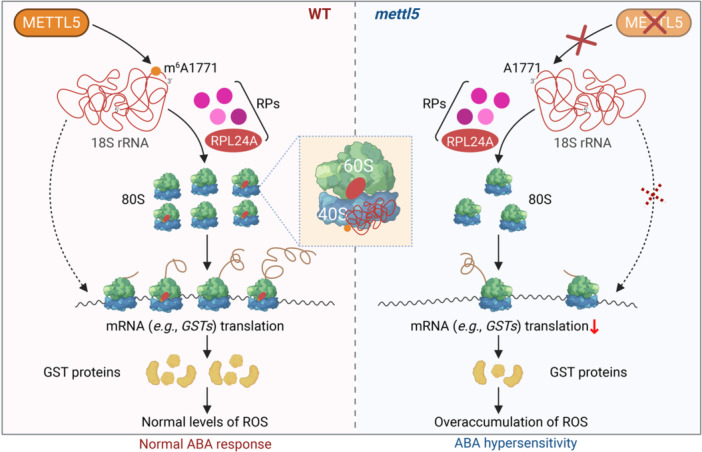

To the Editor,

RNA modifications play central roles in influencing gene expression and cellular function, impacting various biological and physiological processes in eukaryotic organisms. RNA modifications occur on various RNA types, including messenger RNA (mRNA) and ribosomal RNA (rRNA). *N* ^6^‐methyladenosine (m^6^A) has emerged as one of the most widespread and extensively studied modifications [1]. In plants, the mechanisms involved in writing, removing, and interpreting the dynamic mRNA m^6^A methylation have been extensively examined in various development and environmental contexts [2, 3], whereas knowledge of rRNA m^6^A modification is rather limited.

18S rRNA serves as the structural rRNA component for the small subunit (SSU, 40S) in eukaryotic cytoplasmic ribosomes [4, 5]. In human, 18S rRNA carries a single m^6^A modification at A_1832_ mediated by METTL5, which is essential for ribosome function and translation regulation [6−8]. Recently, the cryo‐EM structure of the translating *Nicotiana tabacum* 80S ribosome reveals an m^6^A_1771_ modification in 18S rRNA, which forms a noncanonical base pairing with Cm1645, likely contributing to maintaining the proper positioning of mRNA [6]. However, the mechanisms of m^6^A_1771_ deposition and its role in translation remain largely unknown in plants.

In this study, we reveal that METTL5 catalyzes the deposition of m^6^A at A_1771_ on 18S rRNA, a modification that promotes the association between 18S rRNA and ribosome protein L24 (RPL24A). This interaction facilitates the assembly of 80S ribosome and global translation of mRNAs, including those encoding detoxifying enzymes such as glutathione S‐transferases (GSTs). In the absence of *METTL5*, impaired translation of *GSTs* results in the excessive accumulation of cellular reactive oxygen species (ROS) levels, leading to hypersensitive phenotypes in response to abscisic acid (ABA). Collectively, our findings highlight the essential role of METTL5‐mediated 18S rRNA m^6^A_1771_ modification in global translation and cellular adaptation to oxidative stress.

## RESULTS AND DISCUSSION

### METTL5 catalyzes the deposition of m^6^A on 18S rRNA

**FIGURE 1:**
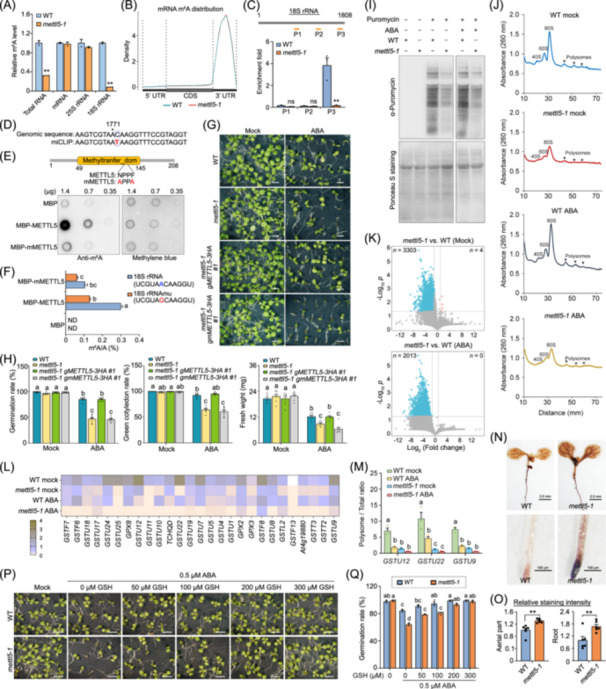
METTL5‐mediated m^6^A modification of 18S rRNA affects ABA response and mRNA translation. (A) Liquid chromatography‐tandem mass spectrometry (LC‐MS/MS) quantification of m^6^A/A ratios in total RNA, mRNA, 25S rRNA, and 18S rRNA. Wild‐type (WT) values were normalized to 1.0. Error bars, mean ± SE; *n* = 2 biological replicates. Asterisks indicate statistically significant differences between WT and *mettl5‐1* plants (**p < 0.01, two‐tailed paired Student's *t* test). (B) Distribution of m^6^A modification sites across different transcript regions (5′ UTR, CDS, and 3′ UTR) in *mettl5‐1* and WT revealed by nanopore direct RNA sequencing. (C) m^6^A‐IP‐qPCR analysis showing abolished m^6^A modification on 18S rRNA in *mettl5‐1*. The upper panel shows amplified regions in m^6^A‐IP‐qPCR. Error bars, mean ± SE; *n* = 3 biological replicates. Asterisk or ns indicate statistically significant or no significant differences between WT and *mettl5‐1* plants (**p < 0.01; ns, *p* > 0.05, two‐tailed paired Student's *t* test). (D) miCLIP assay showing a C‐to‐T transition occurred in 18S rRNA. Asterisk indicates the m^6^A modification site. (E) In vitro m^6^A methylation assay followed by dot blot analysis. The RNA oligo (UCGUAACAAGGU) corresponding to nucleotides 1766‐1777 of 18 rRNA was incubated with MBP, MBP‐METTL5, or MBP‐mMETTL5. m^6^A levels were measured by dot blot analysis. (F) In vitro m^6^A methylation assay followed by LC‐MS/MS analysis. RNA oligo (UCGUAACAAGGU) or mutated RNA oligo (UCGUAGCAAGGU) were incubated with MBP, MBP‐METTL5, or MBP‐mMETTL5, and m^6^A levels were quantified by LC‐MS/MS. ND, not determined. Error bars, mean ± SE. Different letters indicate statistically significant differences (*p* < 0.05, one‐way ANOVA test). (G) ABA sensitivity analysis of *mettl5‐1*, *mettl5‐1 gMETTL5‐3HA*, and *mettl5‐1 gmMETTL5‐3HA* transgenic plants. Seeds were germinated on ½MS medium with or without 0.5 µM ABA, and images were taken after 10 days of growth in the chamber. Bar = 0.5 cm. (H) Analysis of germination rate (left), green cotyledon rate (middle), and total fresh weight of 8 plants (right) of *mettl5‐1*, *mettl5‐1 gMETTL5‐3HA*, and *mettl5‐1 gmMETTL5‐3HA* transgenic plants with or without ABA treatment. Germination rate was calculated 3 days after growth in the chamber, while green cotyledon rate and fresh weight were recorded 10 days after growth. Different letters indicate statistically significant differences (*p* < 0.05, one‐way ANOVA test). (I) Detection of newly synthesized proteins using the SUnSET assay. Six‐day‐old WT and *mettl5‐1* seedlings were mock‐treated or treated with ABA for 5 h and incubated with 50 μM puromycin for 1 h. Total protein was separated on a 15% sodium dodecyl sulfate‐polyacrylamide gel electrophoresis (SDS‐PAGE) gel, and puromycin‐labelled newly synthesized proteins were detected using anti‐puromycin antibody. The RuBisCo large subunit (RbcL), stained with Ponceau S, served as a loading control. (J) Polysome profiling of WT and *mettl5‐1* seedlings under mock or ABA treatment. (K) Volcano plots showing differential gene translation in WT and *mettl5‐1* seedlings under mock or ABA treatment. Grey dotted lines represent the significance thresholds of −Log_10_ *p* > 1.30103 and Log_2_ (Fold change) > 0.584963 or <−0.584963. (L) Translation efficiency of *GST* genes analyzed using our polysome‐seq data. (M) qPCR analysis of the translation efficiency of selected *GST* genes in WT and *mettl5‐1* seedlings under mock or 10 µm ABA treatment for 5 h. Different letters indicate statistically significant differences (*p* < 0.05, one‐way ANOVA test). (N) ROS accumulation visualized by DAB staining in WT and *mettl5‐1* plants. (O) The relative staining intensity of DAB was quantified by Image J. *n* = 6. (P) Growth of WT and *mettl5‐1* plants in the presence of increasing concentrations of GSH under ABA treatment. Photographs were taken after 10 days of growth in the chamber. (Q) Analysis of germination rate of WT and *mettl5‐1* in the presence of GSH under ABA treatment. Bar = 0.5 cm. Germination rate was calculated 3 days after growth. Different letters indicate statistically significant differences (*p* < 0.05, one‐way ANOVA test).

m^6^A modification is catalyzed by a group of RNA methyltransferases characterized by a (D/N)PP(F/Y) motif [7, 8]. To identify novel RNA methyltransferase(s) in *Arabidopsis*, we screened proteins containing this motif and measured m^6^A levels in their mutants (Figure S1A). Among these, *mettl5‐1* mutants exhibited remarkably reduced m^6^A levels in total RNA (Figure 1A and Figure S1B−D). METTL5, the only *Arabidopsis* ortholog of human METTL5, contains the key catalytic motif NPPF (residues 121–124) within its methyltransferase domain, which is well conserved across plant species (Figure S1A,E,F). In *mettl5‐1* mutants, m^6^A levels in 18S rRNA were dramatically reduced, while those in mRNA and 25S rRNA were largely unaffected (Figure 1A). Consistently, another *METTL5* mutant, *mettl5‐2*, also showed greatly reduced m^6^A modification in 18S rRNA (Figures S1B,G). Moreover, we performed nanopore direct RNA sequencing and found that METTL5 had a negligible effect on mRNA m^6^A methylation across the transcriptome (Figure 1B and Figure S2, and Table S1). These results suggest that 18S rRNA is the main methylation target of METTL5.

m^6^A‐immunoprecipitation followed by quantitative PCR (qPCR) detected m^6^A methylation near the 3′ end of 18S rRNA, which was nearly abolished in *mettl5‐1* (Figure 1C). A modified m^6^A individual‐nucleotide cross‐linking and immunoprecipitation (miCLIP) approach, which frequently introduces a C‐to‐T transition at the +1 position of m^6^A site [9], identified a C‐to‐T transition following A_1771_ in 18S rRNA (Figure 1D and Figure S3A), suggesting that A_1771_ is modified. Notably, this site is located in the h44 helix of 18S rRNA (Figure S3B) and corresponds to the m^6^A1832 site identified in animals [10, 11, 12], indicating that METTL5‐mediated m^6^A modification of 18S rRNA is conserved between plants and animals. Our findings align with a recent study showing the deposition of m^6^A to 18S rRNA by METTL5 [13].

To further investigate the methyltransferase activity of METTL5, we performed an in vitro methylation assay and found that METTL5 exhibited methyltransferase activity toward this 18S rRNA fragment (Figure 1E,F, Figure S3C, and Table S2). A catalytically inactive METTL5 variant, generated by mutating the key catalytic residues NPPF to APPA (mMETTL5^121A 124A^; referred to as mMETTL5), showed greatly impaired catalytic activity on 18S rRNA (Figure 1E,F). Additionally, mutating A_1771_ significantly reduced m^6^A levels after incubation with METTL5, suggesting that A_1771_ is the primary m^6^A site mediated by METTL5 (Figure 1F). These results demonstrate that METTL5 deposits m^6^A at the A_1771_ site of 18S rRNA in *Arabidopsis*.

qPCR analysis revealed that *METTL5* was broadly expressed in various tissues, with high expression in shoot apices and young seedlings (Figure S4A). β‐glucuronidase (GUS) staining using *gMETTL5:GUS* transgenic plants showed strong signals in seedlings, inflorescences, and siliques (Figure S4B). Green fluorescent protein (GFP) labelled METTL5 protein GFP‐METTL5 was detected in both nucleus and cytoplasm in *Nicotiana benthamiana* leaf epidermal cells transiently expressing *35S:GFP‐METTL5* and in stable *mettl5‐1 35S:GFP‐METTL5* transgenic plants (Figure S4C−E).

### METTL5 affects ABA signaling

To assess the biological effects of 18S rRNA m^6^A modification, we examined the growth and stress‐response phenotype of *mettl5* mutants. Both *mettl5* mutants did not exhibit obvious growth defects under normal growth conditions throughout the life cycle. Given that m^6^A methylation on mRNAs is crucial for mediating plant response to stress conditions, we next examined whether METTL5 was required for stress response by testing the sensitivity of *mettl5* mutants to ABA. Under ABA treatment, both *mettl5* mutants exhibited markedly decreased seed germination rates, green cotyledon rates, and seedling fresh weight compared to wild‐type (Figure 1G,H and Figure S5), suggesting that *mettl5* mutants are hypersensitive to ABA treatment. Furthermore, the reduced 18S rRNA m^6^A levels and the ABA hypersensitivity phenotype of *mettl5‐1* were fully rescued by *gMETTL5‐3HA* in most transformants examined, but not by the *gmMETTL5‐3HA* construct, which carried the *mMETTL5* variant with impaired m^6^A methyltransferase activity (Figure 1G,H and Figure S6). These results suggest that METTL5‐mediated m^6^A deposition is essential for ABA response.

Although ABA treatment caused significant transcriptome‐wide changes in gene expression in both wild‐type and *mettl5‐1* plants, the overall gene expression profiles of *mettl5‐1* and wild‐type plants were largely similar under mock and ABA treatments (Figure S7A−E, Tables S3 and S4). qPCR analysis further confirmed that the induction of ABA‐responsive genes was comparable between *mettl5‐1* and wild‐type plants under ABA treatment (Figure S7F), despite the observed ABA‐hypersensitivity in *mettl5‐1*. These results suggest that METTL5 modulates ABA response through posttranscriptional regulatory mechanisms, potentially at the translation level.

### METTL5 promotes global translation

METTL5‐mediated m^6^A modification of 18S rRNA did not affect 18S rRNA splicing or abundance (Figure S8). Given that m^6^A_1771_ forms a noncanonical base pairing with Cm1645 in the cryo‐EM structure, likely contributing to the proper positioning of mRNA on the ribosome [6], we hypothesized that METTL5‐mediated m^6^A modification might influence the function of 18S rRNA in translation. To this end, we first performed a SUnSET (surface sensing of translation) assay, in which puromycin incorporation into newly synthesized proteins was used to evaluate the translation activity in wild‐type and *mettl5‐1* plants under mock and ABA treatment conditions. We found a notable reduction in global translation levels in *mettl5‐1* mutants compared to wild‐type, under both conditions (Figure 1I). Polysome profiling analysis further revealed decreased abundance of 80S ribosomes and polysomes in *mettl5* mutants compared to wild‐type plants, under both mock and ABA treatment conditions (Figure 1J). In addition, *gMETTL5*, but not *gmMETTL5*, rescued the defects in translation activity and the reduced abundance of 80S ribosomes and polysomes observed in *mettl5‐1* (Figure S9A,B). Together, these results suggest that METTL5‐mediated 18S rRNA m^6^A modification plays a critical role in maintaining global translation efficiency.

To further elucidate the translational targets affected by METTL5, we performed polysome profiling followed by RNA‐sequencing (polysome‐seq). Consistent with the global translation attenuation revealed by the SUnSET assay, polysome‐seq revealed reduced translation efficiency of 3303 and 2013 transcripts in *mettl5‐1* compared to wild‐type under mock and ABA treatment, respectively (Figure 1K, Tables S5 and S6). Gene Ontology (GO) enrichment analysis of the differentially translated genes under mock conditions identified significant enrichment in stress response pathways, including responses to hypoxia, oxidative stress, ABA, and ABA‐activated signaling pathways (Figure S9C), aligning with the ABA‐hypersensitive phenotype observed in *mettl5‐1*. Similarly, under ABA treatment, GO terms such as response to ABA and ABA signaling pathways were significantly enriched (Figure S9D).

### Excessive ROS accumulation in *mettl5* leads to ABA‐hypersensitivity

Our polysome‐seq data revealed a clear decrease in the translation efficiencies of *GST*s and *Glutathione Peroxidases* (Figure 1L), two gene families involved in detoxifying various toxic compounds, including endogenously derived ROS, and protecting against oxidative damage [14]. qPCR further confirmed the significantly reduced translation efficiencies of three *GSTU* genes (*GSTU9*, *GSTU12*, and *GSTU22*) in *mettl5‐1* mutants compared to wild‐type plants (Figure 1M and Table S7). Consistently, the reduced translation efficiencies of these *GSTU* genes were restored in *mettl5‐1 gMETTL5‐3HA*, but not in *mettl5‐1 gmMETTL5‐3HA* (Figure S9E). A deficiency of detoxifying enzymes may lead to excessive accumulation of ROS, resulting in ABA‐hypersensitivity. We thus assessed ROS levels in wild‐type and *mettl5‐1* plants using 3,3′‐diaminobenzidine (DAB) staining. *mettl5‐1* mutants accumulated significantly higher levels of ROS in root tips and leaves compared to wild‐type (Figure 1N,O), and this phenotype was rescued by *gMETTL5*, not by *gmMETTL5* (Figure S9F).

Since GSTs are involved in maintaining redox homeostasis by detoxifying ROS through conjugation with glutathione (GSH), we proceeded to examine whether the ABA‐hypersensitive phenotype of *mettl5‐1* could be rescued by the exogenous addition of GSH, which restores the redox balance and likely compensates for the reduced GST activity. The addition of GSH does not affect germination or growth under mock conditions (Figure S10A), but increasing concentrations of GSH gradually suppressed the growth inhibition caused by ABA in *mettl5‐1* plants (Figure 1P,Q and Figure S10B). Taken together, these results suggest that excessive ROS accumulation in *mettl5*, resulting from reduced translation efficiency of *GSTs*, leads to ABA‐hypersensitivity.

### Binding of RPL24A to m^6^A_1771_‐modified 18S rRNA regulates translation

**FIGURE 2:**
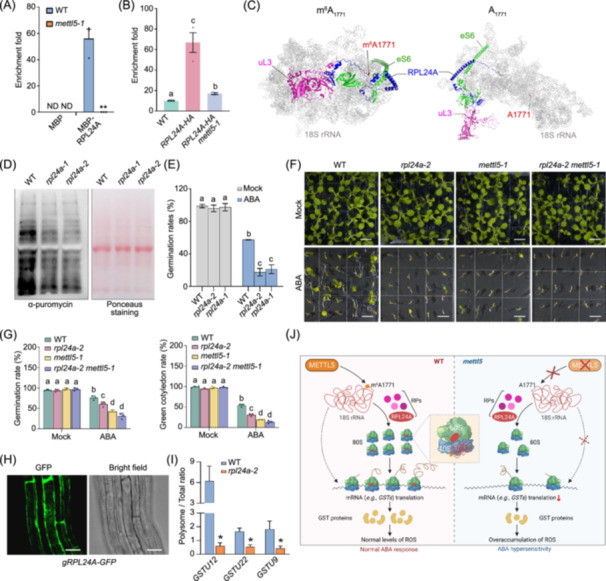
Association of RPL24A with m^6^A‐modified 18S rRNA is required for mRNA translation and ABA response. (A) The affinity between RPL24A and 18S rRNA is decreased in *mettl5‐1* mutants. RNA immunoprecipitation (RIP) was conducted with MBP and MBP‐RPL24A, followed by qPCR analysis using primers against 18S rRNA. (B) RIP‐qPCR detecting the binding of RPL24A with 18S rRNA. (C) Alpha‐fold prediction of the association between RPL24A, eS6 (At5g10360), uL3 (At1g43170), and 18S rRNA with (left) and without (right) m^6^A modification at the nucleotide 1771. (D) SUnSET assay detecting the global newly synthesized proteins. Six‐day‐old WT and *rpl24a* seedlings were treated with ABA for 5 h and then incubated with 50 µM puromycin for 1 h. Total protein was separated on a 15% SDS‐PAGE gel, and the newly synthesized proteins labelled with puromycin were detected using anti‐puromycin antibody. RbcL served as a loading control. (E) Germination rate of *rpl24a* mutants under mock and ABA treatment. Germination rate was calculated 3 days after growth in chamber. (F) ABA sensitivity analysis of *rpl24a‐2* and *rpl24a‐2 mettl5‐1* mutants. Seeds were germinated on ½MS medium with or without 0.5 µM ABA, and images were taken after 10 days of growth. Bar = 0.5 cm. (G) Analysis of germination rate (left) and green cotyledon rate (right) of *rpl24a‐2* and *rpl24a‐2 mettl5‐1* under mock or ABA treatment. Germination rate was calculated 3 days after growth, while green cotyledon rate and fresh weight were recorded after 10 days of growth. Different letters indicate statistically significant differences (*p* < 0.05, one‐way ANOVA test). (H) Localization of RPL24A‐GFP in a *gRPL24A‐GFP* transgenic plant. Bar = 20 μm. (I) qPCR analysis of the translation efficiency of selected *GST* genes in WT and *rpl24a‐2* seedlings under mock or 10 µm ABA treatment for 5 h. Asterisks indicate statistically significant differences between WT and *rpl24‐2* plants (*p < 0.05, two‐tailed paired Student's *t*‐test). (J) A model depicting METTL5‐mediated m^6^A_1771_ of 18S rRNA in protein translation and ABA response. METTL5 catalyzes the m^6^A methylation at A_1771_ in 18S rRNA, a modification essential for its association with the ribosomal protein RPL24A, facilitating the assembly of 80S ribosome. This facilitates the translation of mRNAs encoding the detoxifying GST enzymes, thereby maintaining normal ROS levels and ensuring proper ABA responses. In *mettl5* mutants, the absence of m^6^A_1771_ compromises RPL24A incorporation and ribosome assembly, impairing the translation of *GSTs*. This results in ROS excessive accumulation and hypersensitivity to ABA. RPs, ribosomal proteins. Created with Biorender.

18S rRNA m^6^A_1771_ is located in the h44 loop of 18S rRNA (Figure S3B) [6]. The h44 region of 18S rRNA interacts with the large ribosomal subunit protein RPL24 (also known as eL24), forming the eB13 inter‐subunit bridge that plays an important role in ribosome function [15, 16]. Thus, we proceeded to investigate whether this modification affects its affinity to RPL24A. An in vitro RNA immunoprecipitation assay revealed that RPL24A bound to 18S rRNA in wild‐type plants, whereas this binding was abolished in *mettl5‐1* mutants (Figure 2A). An in vivo RNA immunoprecipitation followed by qPCR (RIP‐qPCR) analysis confirmed the significantly reduced association between RPL24A and 18S rRNA in *mettl5‐1* mutant (Figure 2B), indicating that m^6^A_1771_ is indispensable for the interaction between RPL24A and 18S rRNA. This was further supported by Alpha‐fold predictions, which modeled the eB13 inter‐subunit bridge formed by the ribosome subunits and 18S rRNA, demonstrating a stronger association between RPL24A and m^6^A_1771_‐modified 18S rRNA (Figure 2C). Interestingly, another subunit in the eB13 bridge, eS6 (also known as RPS6), also exhibited reduced association with 18S rRNA in *mettl5‐1* mutants (Figure 2C and Figure S11), further supporting the essential role of m^6^A_1771_ in ribosome assembly.

To further explore the association of RPL24A and 18S rRNA in translation regulation, we obtained two *rpl24a* mutants (Figure S12). Similar to *mettl5* mutants, both *rpl24a* mutants exhibited notable decreases in global translation levels and reduced seed germination rates compared to wild‐type plants upon ABA treatment (Figure 2D,E). *rpl24a‐1* did not further enhance the ABA‐hypersensitive phenotypes of *mettl5‐1* (Figure 2F,G), supporting the notion that METTL5 and RPL24A act in the same genetic pathway in mediating ABA response. Additionally, RPL24A was localized mainly in the cytoplasm (Figure 2H). Moreover, similar to METTL5, RPL24A mediated the translation efficiency of *GST* genes (Figure 2I). Together, these results demonstrate that the interaction between m^6^A_1771_ of 18S rRNA and RPL24A is critical for translation regulation and ABA response.

In this study, we have demonstrated that METTL5‐mediated m^6^A_1771_ of 18S rRNA modulates protein translation and ABA response (Figure 2J). METTL5 catalyzes the m^6^A methylation at A_1771_ in 18S rRNA, a modification essential for the interaction between 18S rRNA and RPL24A, thereby facilitating the assembly of 80S ribosome. METTL5 promotes the translation of mRNAs encoding the detoxifying GST enzymes, thereby maintaining normal ROS levels and ensuring proper ABA responses. In *mettl5* mutants, the absence of m^6^A_1771_ affects RPL24A incorporation and ribosome assembly, impairing the translation of *GSTs*. This results in ROS excessive accumulation and hypersensitivity to ABA. Supporting our findings, a recent study reported that METTL5‐mediated m^6^A_1771_ modification of 18S rRNA facilitates global translation, and regulates blue light‐responsive hypocotyl growth by influencing the translation of blue light‐related mRNAs, such as *HYH* and *PRR9* [13]. Taken together, both studies collectively demonstrate the critical role of METTL5 in translation regulation, particularly in mediating environmental and developmental responses through 18S rRNA modification. Additionally, *mettl5‐1* exhibited hyper‐sensitivity to salt stress (Figure S13), implying a broader role of METTL5‐mediated 18S rRNA m^6^A modification in helping plant cope with stress and adapt to diverse environmental conditions. Moreover, silencing of *NbMETTL5* via VIGS (virus‐induced gene silencing) in *Nicotiana benthamiana* resulted in reduced m^6^A on 18S rRNA and a marked decrease in global translation activity (Figure S14), suggesting a conserved role of METTL5‐mediated 18S rRNA m^6^A modification in regulating translation across plant species.

Additionally, in human, METTL5 forms a complex with TRMT112 to regulate mRNA translation via 18S rRNA m^6^A modification [10, 12, 17, 18]. TRMT112 serves as an essential cofactor that stabilizes METTL5 and enables its catalytic activity for m^6^A methylation of 18S rRNA [18]. However, in *Arabidopsis*, METTL5 does not interact with TRM112A or TRM112B (also known as SMO2) [19], and mutations in *TRM112B*, the major *TRM112* isoform expressed in seedlings, do not influence 18S rRNA m^6^A methylation (Figure S15). Moreover, different from METTL5 (Figure S8), *trm112b* mutants exhibit aberrant pre‐rRNA processing [19]. These findings highlight a divergence in the functional mode of METTL5 between plants and animals, despite their conserved role in methylating 18S rRNA. Whether plant METTL5 requires additional factors to deposit m^6^A on 18S rRNA remains an open question for further investigation.

## CONCLUSION

Our findings uncover a mechanism by which rRNA epitranscriptomic regulation controls mRNA translation and mediates plant responses to environmental conditions, providing insights into how epitranscriptomic marks function as fundamental regulatory systems of gene expression and mediate plant adaptation to fluctuating environments through translational settings.

## METHODS

Detailed procedures for the experiment and data analysis are available in the Supporting Information.

## AUTHOR CONTRIBUTIONS

**Ping Li**: Data curation; formal analysis; validation; writing—original draft; methodology. **Yu Zhang**: Data curation. **Songyao Zhang**: Formal analysis. **Jinqi Ma**: Data curation. **Sheng Fan**: Data curation. **Lisha Shen**: Conceptualization; supervision; funding acquisition; project administration; writing—review and editing.

## CONFLICT OF INTEREST STATEMENT

The authors declare no conflicts of interest.

## ETHICS STATEMENT

No animals or humans were involved in this study.

## Supporting information


**Figure S1:** Characterization of METTL5.
**Figure S2:** Differential m^6^A modification rate analyzed by nanopore direct RNA sequencing.
**Figure S3:** 18S rRNA is modified with an m^6^A at the A_1771_ site.
**Figure S4:** *METTL5* expression pattern and subcellular localization.
**Figure S5:** METTL5 affects ABA response.
**Figure S6:** *gMETTL5‐3HA* but not *gmMETTL5‐3HA* rescues the ABA hypersensitivity of *mettl5‐1*.
**Figure S7:** *METTL5* has minor effects on global gene transcription.
**Figure S8:** METTL5‐mediated m^6^A modification does not affect rRNA processing and abundance.
**Figure S9:** *gMETTL5*, but not *gmMETTL5*, restores the defects in translational efficiency observed in *mettl5‐1*.
**Figure S10:** Exogenous addition of GSH does not affect seed germination or growth of *mettl5‐1* mutants.
**Figure S11:** The affinity between eS6 and 18S rRNA is decreased in *mettl5‐1* mutants.
**Figure S12:** Characterization of *RPL24A* mutants.
**Figure S13:** *mettl5* mutants are hypersensitive to salt stress.
**Figure S14:** Silencing of *NbMETTL5* reduces 18S rRNA m^6^A levels and global translation in *N. benthamiana*.
**Figure S15:** TRM112A/B does not interact with METTL5.

**Table S1:** Differentially modified genes in *mettl5‐1* vs. wild type (DRM > 0.3 and <−0.3).
**Table S2:** List of primers used in this study.
**Table S3:** Differentially expressed genes in *mettl5‐1* vs. wild type under mock conditions.
**Table S4:** Differentially expressed genes in *mettl5‐1* vs. wild type under ABA treatment.
**Table S5:** Genes exhibiting differential translation efficiency in *mettl5‐1* vs. wild type under mock conditions.
**Table S6:** Genes exhibiting differential translation efficiency in *mettl5‐1* vs. wild type under ABA treatment.
**Table S7:** Polysome to total RNA ratios of *GST* gene expression, as shown in Figure 1M.

## Data Availability

The data that support the findings of this study are openly available in PRJNA1227307 and PRJNA1226565 at https://bigd.big.ac.cn/gsa/index.jsp. RNA‐seq, polysome‐seq, and m^6^A nanopore‐seq data generated in this study were deposited into the sequence read archive (SRA) of the National Center for Biotechnology Information (NCBI) under the accession numbers: PRJNA1227307 (RNA‐seq, https://www.ncbi.nlm.nih.gov/bioproject/PRJNA1227307), PRJNA1226565 (polysome‐seq, https://www.ncbi.nlm.nih.gov/bioproject/PRJNA1226565), and (https://www.ncbi.nlm.nih.gov/bioproject/PRJNA1264749). Supplementary materials (methods, figures, tables, graphical abstract, slides, videos, Chinese translated version, and update materials) may be found in the online DOI or iMeta Science http://www.imeta.science/.
