# Supplementary material for: METTL5‐mediated 18S rRNA m6A modification enhances ribosome assembly and ABA response in Arabidopsis
Source: Imeta. 2025 Jun 13;4(4):e70055. doi: 10.1002/imt2.70055 (PMC12371265; doi:10.1002/imt2.70055)
Supplement: Supplementary file 1 — Figure S1: Characterization of METTL5. Figure S2: Differential m6A modification rate analyzed by nanopore direct RNA sequencing. Figure S3: 18S rRNA is modified with an m6A at the A1771 site. Figure S4: METTL5 expression pattern and subcellular localization. Figure S5: METTL5 affects ABA response. Figure S6: gMETTL5‐3HA but not gmMETTL5‐3HA rescues the ABA hypersensitivity of mettl5‐1. Figure S7: METTL5 has minor effects on global gene transcription. Figure S8: METTL5‐mediated m6A modification does not affect rRNA processing and abundance. Figure S9: gMETTL5, but not gmMETTL5, restores the defects in translational efficiency observed in mettl5‐1. Figure S10: Exogenous addition of GSH does not affect seed germination or growth of mettl5‐1 mutants. Figure S11: The affinity between eS6 and 18S rRNA is decreased in mettl5‐1 mutants. Figure S12: Characterization of RPL24A mutants. Figure S13: mettl5 mutants are hypersensitive to salt stress. Figure S14: Silencing of NbMETTL5 reduces 18S rRNA m6A levels and global translation in N. benthamiana. Figure S15: TRM112A/B does not interact with METTL5. [file IMT2-4-e70055-s001.docx]

**Supporting information to**

**METTL5-mediated 18S rRNA m^6^A modification enhances ribosome assembly and ABA response in *Arabidopsis***

**Running title:** 18S rRNA m^6^A modification

Ping Li^1^, Yu Zhang^1^, Songyao Zhang^2^, Jinqi Ma^1,2^, Sheng Fan^1^, Lisha Shen^1,2*^

^1^Temasek Life Sciences Laboratory, National University of Singapore, Singapore 117604, Singapore

^2^Department of Biological Sciences, National University of Singapore, Singapore 117543, Singapore

*Correspondence: [lisha@tll.org.sg](mailto:lisha@tll.org.sg) (Lisha Shen)

**METHODS**

**Plant materials and growth conditions**

*Arabidopsis thaliana* plants (Col-0 background) were grown on soil or Murashige and Skoog (MS) plates under long day conditions (16 h light / 8 h dark) at 22 ± 2℃. The *mettl5-1*, *mettl5-2*, and *rpl24a-2* mutants were generated using CRISPR/Cas9. Various transgenic plants in wild-type or *mettl5-1* mutant backgrounds were generated using *Agrobacterium tumefaciens*-mediated transformation via floral dipping [1]. To assess ABA-sensitive phenotypes, *Arabidopsis* seeds were surface-sterilized with 10% sodium hypochlorite and sown on ½ MS plates supplemented with various concentrations of ABA.

**Plasmid construction**

To construct CAS9-*METTL5*-1 and CAS9-*METTL5*-2, the synthesized sgRNA oligos were cloned into a modified pENTR vector containing the CAS9-sgRNA cassette [2]. To generate *gMETTL5-3HA* and *gMETTL5-GUS*, the genomic region of *METTL5* was amplified using gMETTL5-GW-F and METTL5-GW-R and cloned into the pDONR207 vector through a BP reaction. The *gMETTL5* fragment was then recombined into pGWB13 and pGWB3 vectors to generate *gMETTL5-3HA* and *gMETTL5-GUS*, respectively, via Gateway LR recombination (Invitrogen). Similarly, the coding region of *METTL5* was amplified and cloned into the pDONR207 vector, and recombined into the pMDC43 vector to generate *35S:METTL5-GFP*. To obtain the CAS9-RPL24A plasmid, the synthesized sgRNA oligos were cloned into a modified pC1300-gRNA-Cas9 vector. To generate the *gRPL24A-GFP* plants, the genomic fragment of *RPL24A* was cloned into a modified pCAMBIA1305 vector with GFP tag. Primers used for plasmid construction are listed in Table S7.

**Expression analysis**

Total RNA was extracted using the FavorPrep™ Plant Total RNA Mini Kit (Favorgen) and reverse-transcribed using the M-MLV Reverse Transcriptase (Promega) according to manufacturers’ instructions. Quantitative real-time PCR was performed on three biological replicates using QuantStudio^TM^ 5 Real-Time PCR System (Applied Biosystems) with Taq Pro Universal SYBR qPCR Master Mix (Vazyme). Expression of *TUB2* served as an internal control. Relative expression levels of genes were determined as previously described [3]. Primers used for gene expression analysis are listed in Table S7. GUS staining of *gMETTL5-GUS* transgenic plants was conducted as previously described [3].

**RNA sequencing**

For RNA sequencing, 6-day-old seedlings of wild-type and *mettl5-1* grown on MS plates were submerged in liquid ½ MS medium for 3 hours. The medium was then replaced with ½ MS medium with or without ABA for 5 h, after which the seedlings were harvested for total RNA extraction using the FavorPrep™ Plant Total RNA Mini Kit (FAVORGEN). RNA quality and quantity were assessed with an Agilent Bioanalyzer 2100 system. rRNA was removed using the Ribo-Zero rRNA Removal Kit (Eipcentre). The NEBNext Ultra II Directional RNA Library Prep Kit for Illumina (NEB) was used for library construction, and sequencing was performed on the Illumina Hiseq 2500 platform. RNA-seq data were analyzed as previously described [4].

**Analysis of m^6^A level by dot blot**

18S and 25S rRNA were purified from total RNA by gel electrophoresis followed by the Zymoclean Gel RNA Recovery Kit (Zymo Research). Dot blot analysis was conducted as previously described [5,6]. In brief, denatured RNA was applied to a Hybond-N+ membrane and crosslinked using a Stratalinker 2400 UV Crosslinker (Stratagene). The membrane was then washed with 1 × PBST buffer for 5 min, followed by blocking with 5% non-fat milk in 1 × PBST for 1 h at room temperature. The membrane was incubated with anti-m^6^A antibody (Synaptic Systems) overnight at 4°C, then with horseradish-peroxidase-conjugated anti-rabbit IgG secondary antibody (Santa Cruz). Subsequently, the membrane was visualized with an ECL Western Blotting Detection Kit (Thermo) in a ChemiDoc Touch Imaging System (Bio-rad).

**Analysis of m^6^A level by liquid chromatography-tandem mass spectrometry (LC-MS/MS)**

Measurement of m^6^A levels by LC-MS/MS was performed as previously described [5]. rRNA and mRNA were purified from total RNA using RNA gel purification and the Dynabeads™ mRNA Purification Kit (Invitrogen), respectively. The RNA was then digested into single ribonucleosides, purified with chloroform, and analyzed by LC-MS/MS on a SCIEX QTRAP 6500 spectrometer. Multiple reaction monitoring mode was used to detect A and m^6^A, with mass transitions at 268.0 to 136.0 and 282.0 to 150.1, respectively.

**m^6^A-immunoprecipitation-qPCR (m^6^A-IP-qPCR)**

m^6^A-IP-qPCR was performed as reported [7,8]. Total RNA was chemically fragmented into ~200-nucleotide-long fragments and incubated with anti-m^6^A antibody (Synaptic Systems) in 1 × IP buffer (10 mM Tris-HCl pH 7.4, 150 mM NaCl, 0.1% Igepal CA-630) supplemented with RNasin Plus RNase inhibitor (Promega) for 2 h at 4°C with gentle rotation. This solution was then incubated with BSA-bound Protein A/G Plus Agarose (Santa Cruz) for 2 h at 4°C with gentle rotation. After extensive washing, the bound RNA was eluted from the beads with 1 × IP buffer supplemented with *N*^6^-methyladenosine 5′-monophosphate sodium salt (Sigma) and precipitated with ethanol. Input and immunoprecipitated RNAs were then reverse transcribed using random hexamers (Invitrogen) with M-MLV Reverse Transcriptase (Promega). The relative enrichment of each fragment was determined by quantitative real-time PCR and calculated as previously described [7], with *TUB2* used as an internal control. The primers used for m^6^A-IP-qPCR analysis are listed in Table S7.

**UV cross-linking and immunoprecipitation for m^6^A (m^6^A-CLIP)**

m^6^A-CLIP was performed as previously described with some modifications [9]. Total RNA was incubated with anti-m^6^A antibody in binding buffer (50 mM Tris-HCl pH 7.4, 150 mM sodium chloride, 0.5% NP-40) supplemented with RNase inhibitor (Promega) for 2 h at 4°C with gentle rotation. The RNA:antibody solution was then cross-linked twice at 150 mJ/cm^2^ (254 nm wavelength) in a Stratalinker UV Crosslinker. Protein A/G magnetic beads were added into this solution and incubated at 4°C for 4 h with rotation. After incubation, the beads were washed twice with high salt wash buffer (50 mM Tris-HCl pH 7.4, 1 M sodium chloride, 1 mM EDTA, 1% NP-40, 0.5% sodium deoxycholate, 0.1% sodium dodecyl sulfate) and once with wash buffer (20 mM Tris-HCl pH 7.4, 10 mM magnesium chloride, 0.2% Tween-20). Subsequently, the beads were resuspended in wash buffer supplemented with Proteinase K and incubated at 55°C for 15 min, followed by RNA extraction using phenol-chloroform. The purified RNA was reverse transcribed using the M-MLV Reverse Transcriptase (Promega) according to the manufacturer’s instructions. Fragments of 18S rRNA were amplified and ligated into the pGEM^®^-T vector (Promega) followed by sequencing.

***In vitro* methylation assay**

*In vitro* methylation assay was performed as previously described [5,10,11]. Full-length coding sequences of METTL5 and mMETTL5(METTL5^APPA^) were cloned into pMAL-c2x vector (NEB). MBP, MBP-METTL5, and MBP-mMETTL5 recombinant proteins were expressed in *Escherichia coli* Rosetta (DE3) cells and purified with Amylose Resin (NEB). Recombinant MBP, MBP-METTL5 or MBP-mMETTL5 proteins were incubated with the synthesized RNA probe (UCGUAACAAGGU) or mutated RNA probe (UCGUAGCAAGGU) in binding buffer (50 mM Tris-HCl pH 8.0, 2 mM MgCl_2_, 5 mM KCl, 2 mM DTT, 3 mM SAM, 40 U RNase inhibitor) at 30°C for 2 h. After incubation, RNA was purified with chloroform and subjected to dot blot or LC-MS for m^6^A level determination.

**Polysome profiling**

Total polysomes were extracted as previously described with some modifications [12,13]. Briefly, seedlings (~1 g) were ground in liquid nitrogen and the powder was resuspended in 10 mL of lysis buffer (25 mM Tris-HCl pH 7.4, 150 mM NaCl, 5 mM MgCl_2_, 1 mM DTT, 1 mM PMSF, 40 U/mL RNase inhibitor, 1% sodium deoxycholate, 0.5% NP-40). The slurry was filtered through cell strainers, and the filtered liquid was centrifuged at 20,000 × rpm for 20 min at 4°C. A 200 mL aliquot of the supernatant was collected as the total RNA control. For polysome profiling, supernatants were loaded onto a 10−50% sucrose step gradient and centrifuged at 200,000 × g in an SW41 Ti rotor (Beckman Coulter) for 3 h. Fractions were collected from the top to the bottom of the gradient with continuous monitoring of absorbance at 260 nm. Polysomal fractions were pooled separately for RNA extraction.

**SUnSET assay**

The SUnSET assay was performed according to a previously published protocol with minor modifications [14,15]. Briefly, 6-day-old seedlings were incubated in liquid MS medium with or without 10 mM ABA for 5 h, followed by the addition of 50 mM puromycin for an additional 1 h. Samples were collected and ground into powder in liquid nitrogen. Total protein was then extracted, separated by SDS-PAGE, and detected using an anti-puromycin antibody (ZMS1016 Sigma). The Rubisco large subunit (rbcL), stained with Ponceau S, served as the loading control.

**Yeast two-hybrid assay**

The coding sequences of METTL5, TRM112A, and TRM112B were amplified and cloned into pGADT7 (AD) or pGBKT7 (BD) vectors (Clontech). Different combinations of various AD and BD vectors were co-transformed into AH109 cells using the Yeastmaker Yeast Transformation system 2 (Clontech). Subsequently, the transformed yeast cells were grown on SD-Leu/-Trp and SD-Ade/-His/-Leu/-Trp medium.

**Protein expression in *N. benthamiana* cells**

To observe the localization of *35S:GFP*, *35S:GFP-METTL5*, *35S:TRM112A-GFP*, and *35S:TRM112B-GFP*, *Agrobacterium* suspension containing the respective vectors in infiltration buffer (10 mM MES pH 5.6, 10 mM MgCl_2_, 100 µM acetosyringone) were injected into the abaxial surface of *N. benthamiana* leaves with syringes. After incubation for 2 days, the signals were observed under a confocal microscope.

**Nanopore direct RNA sequencing and data analysis**

Total RNA was extracted from 7-day-old seedlings from WT and *mettl5-1* using the RNeasy Plus Mini kit (QIAGEN) according to the manufacturer’s instructions. mRNA was then isolated using Dynabeads mRNA purification kit (Invitrogen). RNA library was prepared using the nanopore direct RNA sequencing kit (SQK-RNA004, Oxford Nanopore Technologies) according to the manufacturer’s instructions. Briefly, 150 ng of mRNA was ligated with RT Adapter using T4 DNA ligase (NEB M0202M) and then was reverse transcripted using Induro Reverse Transcriptase (NEB M0681L). After the purification with Agencourt RNAClean XP beads (Beckman Coulter), the RT-RNA sample was then ligated with RNA ligation Adapter using T4 DNA ligase (NEB M0202M). The RNA library was purified with Agencourt RNAClean XP beads (Beckman Coulter) and eluted with RNA Elution Buffer. The resulting RNA libraries were loaded into the primed FLO-PRO004RA flow cells using a PromethION 2 solo sequencer with MinKNOW acquisition software according to the manufacturer’s instructions.

m^6^A sites were identified using Dorado (v 0.8.2) and Modkit (v 0.4.2) from Oxford Nanopore Technologies. Briefly, the “m^6^A” model of “rna004_130bps_hac@v5.0.0” was adopted for Dorado for simultaneous base calling and m^6^A detection for each read. The identified modifications were subsequently piled up using Modkit with parameters “--filter-threshold 0.75 --mod-thresholds a:0.9”. To further refine the results, R (v 4.3.3) was used to filter reliable m^6^A sites based on the following criteria: 1) the reference nucleotide of the position is “A”; 2) at least five modified reads are present at the position; 3) the modification rate (*i.e.*, fraction of modified reads) on the position is at least 30%; and 4) these criteria are met consistently across all three replicates.

**Virus-induced gene silencing**

A 320 bp fragment of *NbMETTL5* (Niben101Scf04103g09013) was amplified from *N. benthamiana* leaf cDNA and cloned into the TRV2 vector to generate *TRV2-NbMETTL5*. The vector was then introduced into *Agrobacterium tumefaciens* strain EHA105 by electroporation. *N. benthamiana* plants at the two-to-three true leaf stage were co-inoculated with the *Agrobacteria* suspensions (OD_600_ = 0.6) containing either *TRV2-NbMETTL5* or *TRV2-GUS* and the helper virus TRV1. Leave samples were harvested 14 days after inoculation for total RNA extraction. Silencing efficiency was assessed by qPCR, measuring *NbMETTL5* expression levels relative to the endogenous control *GAPDH*.

**Statistical analysis**

Statistical tests performed on experimental data and sample sizes are noted in figure legends. All data points were derived from biological replicates and analyzed using GraphPad Prism 10.4.2. For comparisons between two groups, a *t*-test (and nonparametric tests) was employed. For comparisons of data with more than two groups, One-Way ANOVA (and nonparametric or mixed) was used. Multiple comparisons were performed using Tukey’s multiple comparisons test.

**REFERENCES**

1. Clough, Steven J., and Andrew F. Bent. 1998. “Floral dip: a simplified method for Agrobacterium-mediated transformation of Arabidopsis thaliana.” *Plant Journal* 16: 735-743. <https://doi.org/10.1046/j.1365-313x.1998.00343.x>

2. Shen, Lisha. 2023. “Functional interdependence of m^6^A methyltransferase complex subunits in Arabidopsis.” *Plant Cell* 35(6): 1901-1916. <https://doi.org/10.1093/plcell/koad070>

3. Chen, Ying, Shiyong Song, Yinbo. Gan, Lixi Jiang, Hao Yu, and Lisha Shen. 2020. “SHAGGY-like kinase 12 regulates flowering through mediating CONSTANS stability in Arabidopsis.” *Science Advances* 6: eaaw0413. <https://doi.org/10.1126/sciadv.aaw0413>

4. Zhang, Yu, Sheng Fan, Changmei Hua, Zhi Wei Norman Teo, Jian Xuan Kiang, Lisha Shen, et al. 2022. “Phase separation of HRLP regulates flowering time in Arabidopsis.” *Science Advances* 8: eabn5488. <https://doi.org/10.1126/sciadv.abn5488>

5. Xu, Tao, Xiaowei Wu, Chui Eng Wong, Sheng Fan, Yu Zhang, Songyao Zhang, et al. 2022. “FIONA1-mediated m^6^A modification regulates the floral transition in Arabidopsis.” *Advanced Science* 9: e2103628. <https://doi.org/10.1002/advs.202103628>

6. Shen, Lisha, Zhe Liang, and Hao Yu. 2017. “Dot blot analysis of *N*^6^-methyladenosine RNA modification levels.” *Bio Protocol* 7: e2095. <https://doi.org/10.21769/BioProtoc.2095>

7. Shen, Lisha, Zhe Liang, Xiaofeng Gu, Ying Chen, Zhi Wei Norman Teo, Xingliang Hou, et al. 2016. “*N*^6^-methyladenosine RNA modification regulates shoot stem cell fate in Arabidopsis.” *Developmental Cell* 38: 186-200. <https://doi.org/10.1016/j.devcel.2016.06.008>

8. Dominissini, Dan, Sharon Moshitch-Moshkovitz, Mali Salmon-Divon, Ninette Amariglio, and Gideon Rechavi. 2013. “Transcriptome-wide mapping of *N*^6^-methyladenosine by m^6^A-seq based on immunocapturing and massively parallel sequencing.” *Nature Protocols* 8: 176-189. <https://doi.org/10.1038/nprot.2012.148>

9. Roberts, Justin T., Allison M. Porman, and Aaron M. Johnson. 2021. “Identification of m^6^A residues at single-nucleotide resolution using eCLIP and an accessible custom analysis pipeline.” *RNA* 27: 527-541. <https://doi.org/10.1261/rna.078543.120>

10. Rong, Bowen, Qian Zhang, Jinkai Wan, Shenghui Xing, Ruofei Dai, Yuan Li, et al. 2020. “Ribosome 18S m^6^A methyltransferase METTL5 promotes translation initiation and breast cancer cell growth.” *Cell Reports* 33: 108544. <https://doi.org/10.1016/j.celrep.2020.108544>

11. Cui, Xuean, Zhe Liang, Lisha Shen, Qian Zhang, Shengjie Bao, Yuke Geng, et al. 2017. “5-Methylcytosine RNA methylation in Arabidopsis thaliana.” *Molecular Plant* 10: 1387-1399. <https://doi.org/10.1016/j.molp.2017.09.013>

12. Merchante, Catharian, Javier Brumos, Jeonga Yun, Qiwen Hu, Kristina R. Spencer, Paul Enriquez, et al. 2015. “Gene-specific translation regulation mediated by the hormone-signaling molecule EIN2.” *Cell* 163: 684-697. <https://doi.org/10.1016/j.cell.2015.09.036>

13. Wang, Yuan, Shaofang Li, Yonghui Zhao, Chenjiang You, Brandon Le, Zhizhong Gong, et al. 2019. “NAD(+)-capped RNAs are widespread in the Arabidopsis transcriptome and can probably be translated.” *Proceedings of the National Academy of Sciences of the United States of America* 116: 12094-12102. <https://doi.org/10.1073/pnas.1903682116>

14. Chen, Tianyuan, Guoyong Xu, Rui Mou, George H. Greene, Lijing Liu, Jonathan Motley, et al. 2023. “Global translational induction during NLR-mediated immunity in plants is dynamically regulated by CDC123, an ATP-sensitive protein.” *Cell Host & Microbe* 31: 334-342 e335. <https://doi.org/10.1016/j.chom.2023.01.014>

15. Van Hoewyk, Doug 2016. “Use of the non-radioactive SUnSET method to detect decreased protein synthesis in proteasome inhibited Arabidopsis roots.” *Plant Methods* 12: 20. <https://doi.org/10.1186/s13007-016-0120-z>


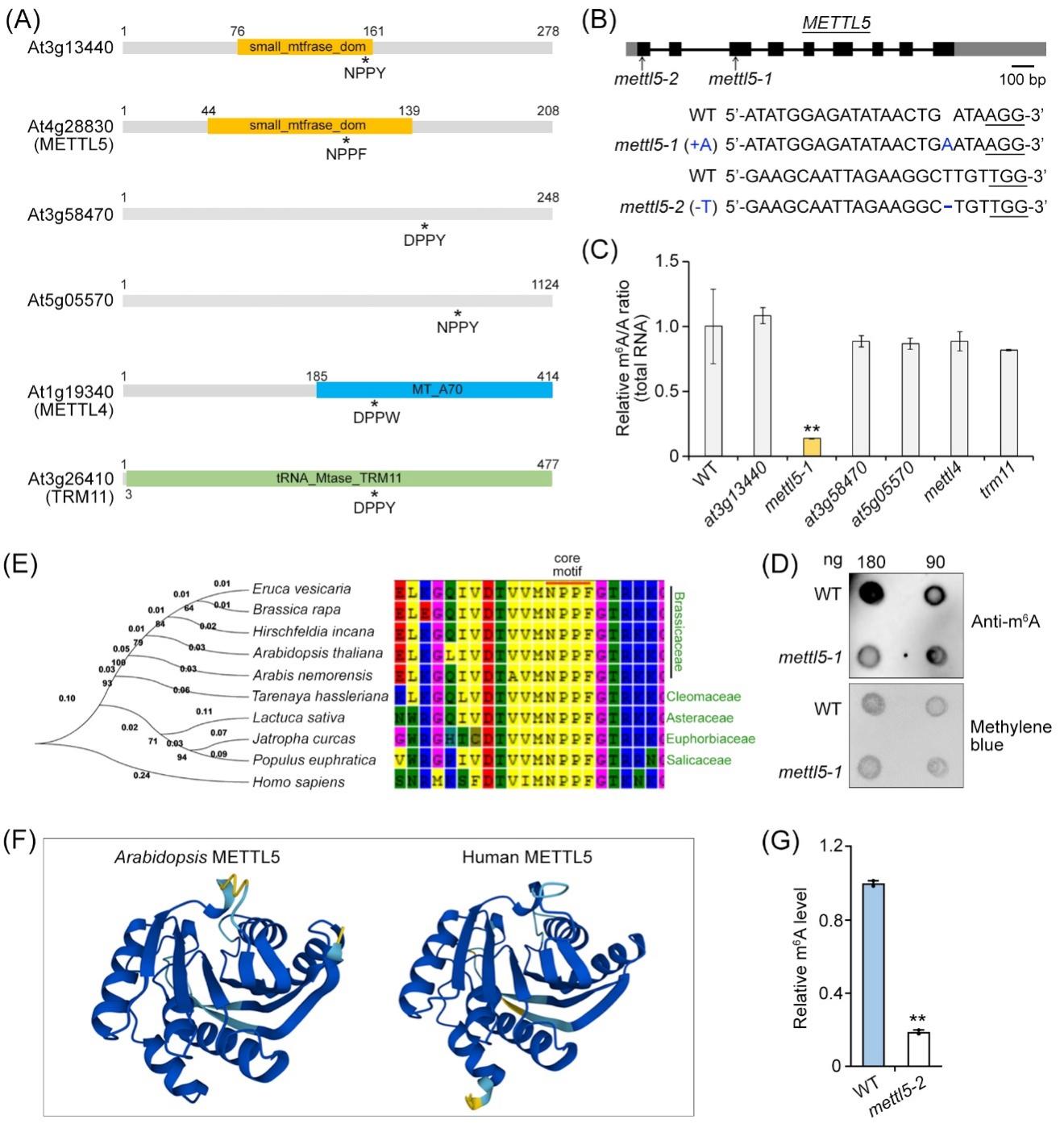
**Figure S1 Characterization of METTL5.** (A) *Arabidopsis* proteins containing (D/N)PP(Y/F) sequences. The positions of (D/N)PP(Y/F) sequences, small_mtfrase_domain, MT_A70 domain, and the tRNA_Mtase_TRM11 domain are indicated. (B) CRISPR/Cas9-mediated mutagenesis of *METTL5*. The upper panel shows the *METTL5* genomic sequence and mutation sites in *mettl5-1* and *mettl5-2*. Grey and black boxes represent untranslated regions (UTRs) and exons in the coding region, respectively, while black lines indicate introns. The lower panel shows alignment of wild-type (WT), *mettl5-1*, and *mettl5-2* sequences containing the CRISRP/Cas9 target sites with underlined protospacer adjacent motifs. *mettl5-1* has a 1-bp insertion (A), and *mettl5-2* has a 1-bp deletion (T). (C) LC-MS/MS quantification of m^6^A/A ratios in total RNA of various mutants. Mutants of *At3g13440*, *METTL4*, and *TRM11* were obtained from ABRC, while mutants of *METTL5*, *At3g58470*, and *At5g05570* were generated using CRISPR/Cas9-mediated mutagenesis. Error bars, mean ± SE; *n* = 2 biological replicates. Asterisks indicate statistically significant differences between WT and *mettl5-1* plants (***p* < 0.01, two-tailed paired Student’s *t* test). (D) Dot blot analysis of total m^6^A levels in *mettl5-1* mutants. Total RNA was isolated from 6-day-old wild-type and *mettl5-1* seedlings. (E) Phylogenetic analysis of METTL5 across various plant species and human, with fragments containing the NPPF motif highlighted. The Neighbor-Joining method was employed, and a bootstrap analysis with 1000 replicates was conducted. Bootstrap values and genetic variations were displayed in the cladogram. The phylogenetic analysis was carried out using MEGA5. (F) AlphaFold predicted protein structure of *Arabidopsis* METTL5 compared to human METTL5. (G) LC-MS/MS quantification of m^6^A/A in 18S rRNA in *mettl5-2*. WT values were normalized to 1.0. Error bars, mean ± SE; *n* = 2 biological replicates. Asterisk indicates statistically significant differences (***p* < 0.01, two-tailed paired Student’s *t* test).


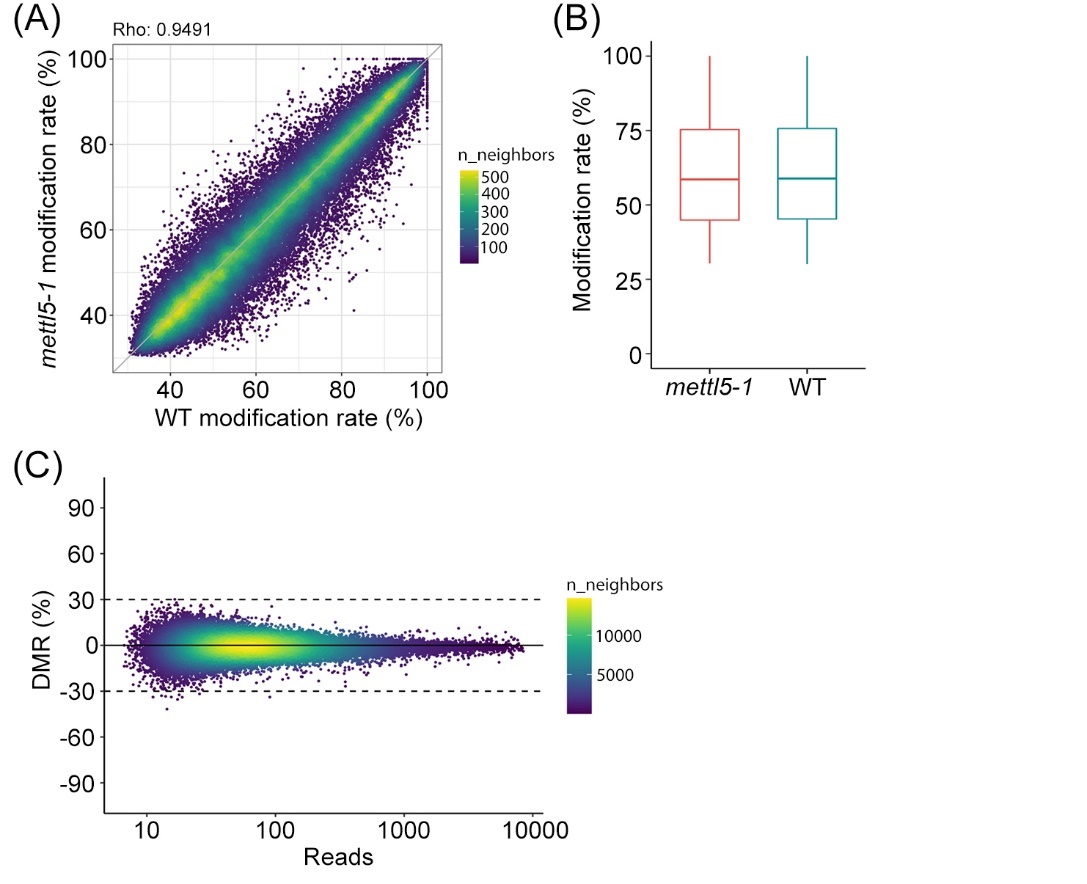


**Figure S2** **Differential m^6^A modification rate analyzed by nanopore direct RNA sequencing.** (A) Scatter plot comparing m^6^A modification rates between *mettl5-1* and WT, showing a high correlation. Color intensity indicates the number of neighboring data points. (B) Boxplot of m^6^A modification rates in *mettl5-1* and WT, showing overall distribution differences. (C) Scatter plot showing differential modification rates (DMR) between *mettl5-1* and WT, with color indicating the number of neighboring data points.


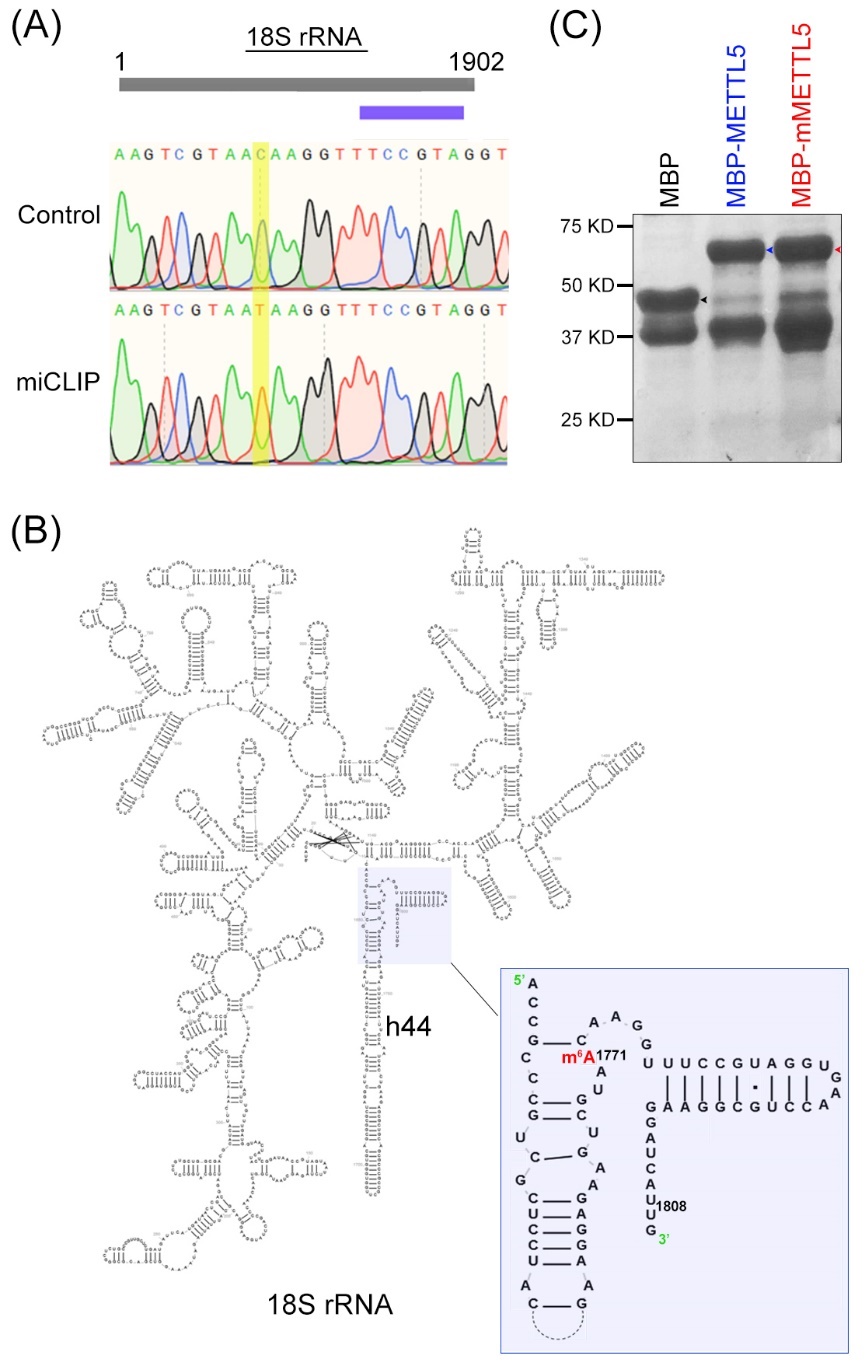


**Figure S3 18S rRNA is modified with an m^6^A at the A_1771_ site.** (A) Chromatography from sanger sequencing showing a C-to-T transition occurred in 18S rRNA detected by the miCLIP assay. (B) The secondary structure of *Arabidopsis* 18S rRNA created based on the RNAcentral database (The RNAcentral Consortium, 2019; <http://www.rna.icmb.utexas.edu>. The position of m^6^A_1771_ was highlighted in the enlarged inset. (C) Expression of recombinant MBP-METTL5 and MBP-mMETTL5 used in the *in vitro* methyltransferase assay. Arrowheads indicate the expected proteins.


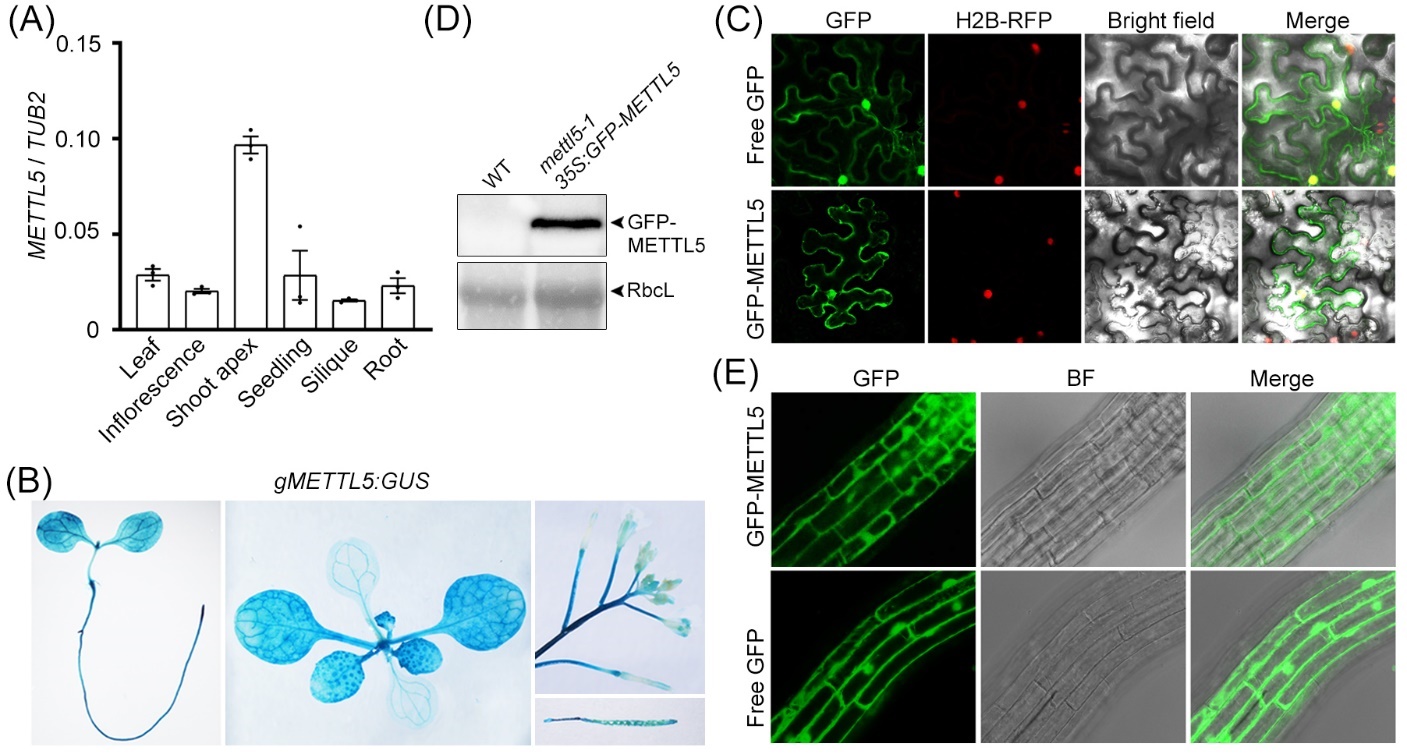
**Figure S4 *METTL5* expression pattern and subcellular localization.** (A) qPCR analysis of *METTL5* expression in various organs. (B) *METTL5* expression patterns in various organs revealed by GUS staining with *gMETTL5-GUS*. From left to right: 5-day-old seedling, 15-day-old seedling, floral bud (top), and silique (bottom). (C) Subcellular localization of GFP-METTL5 in *N. benthamiana* leaf epidermal cells. *35S:GFP* was used a control. H2B-RFP, RFP fluorescence of the nuclear reporter (core histone 2B fused to red fluorescent protein). (D) Immunoblot analysis of GFP-METTL5 expression in *mettl5-1 35S:GFP-METTL5* transgenic plants. RbcL serves as a loading control. (E) Subcellular localization of METTL5-GFP and free GFP in *mettl5-1 35S:GFP-METTL5* and *35S:GFP* transgenic plants, respectively.

**
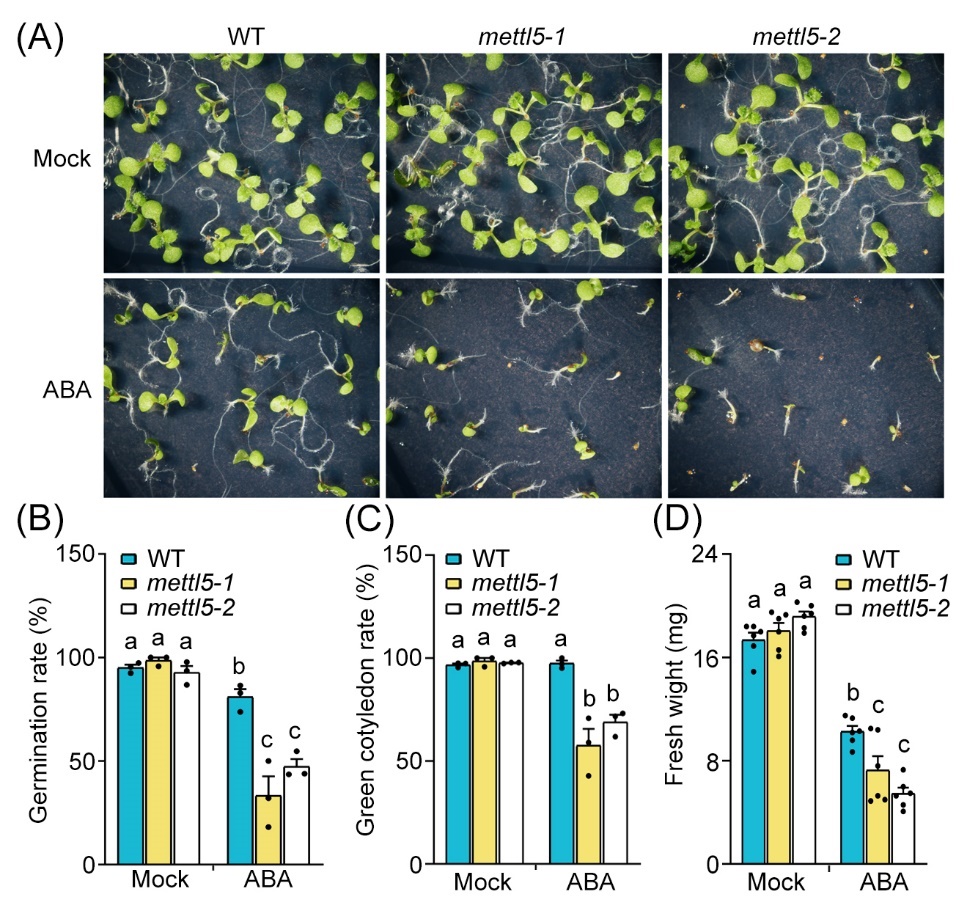
**

**Figure S5 METTL5 affects ABA response.** (A) ABA sensitivity analysis of wild-type (WT) plants and *mettl5* mutants. Seeds were germinated on ½MS medium with or without 0.5 µM ABA, and images were taken 7 days after growth in ½MS medium. (B−D) Analysis of germination rate (B), green cotyledon rate (C), and total fresh weight of 8 plants (D) for WT and *mettl5* plants with or without ABA treatment. Germination rate was calculated 3 days after growth, while green cotyledon rate and fresh weight were recorded after 7 days of growth. Different letters indicate statistically significant differences (*p* < 0.05, one-way ANOVA test).


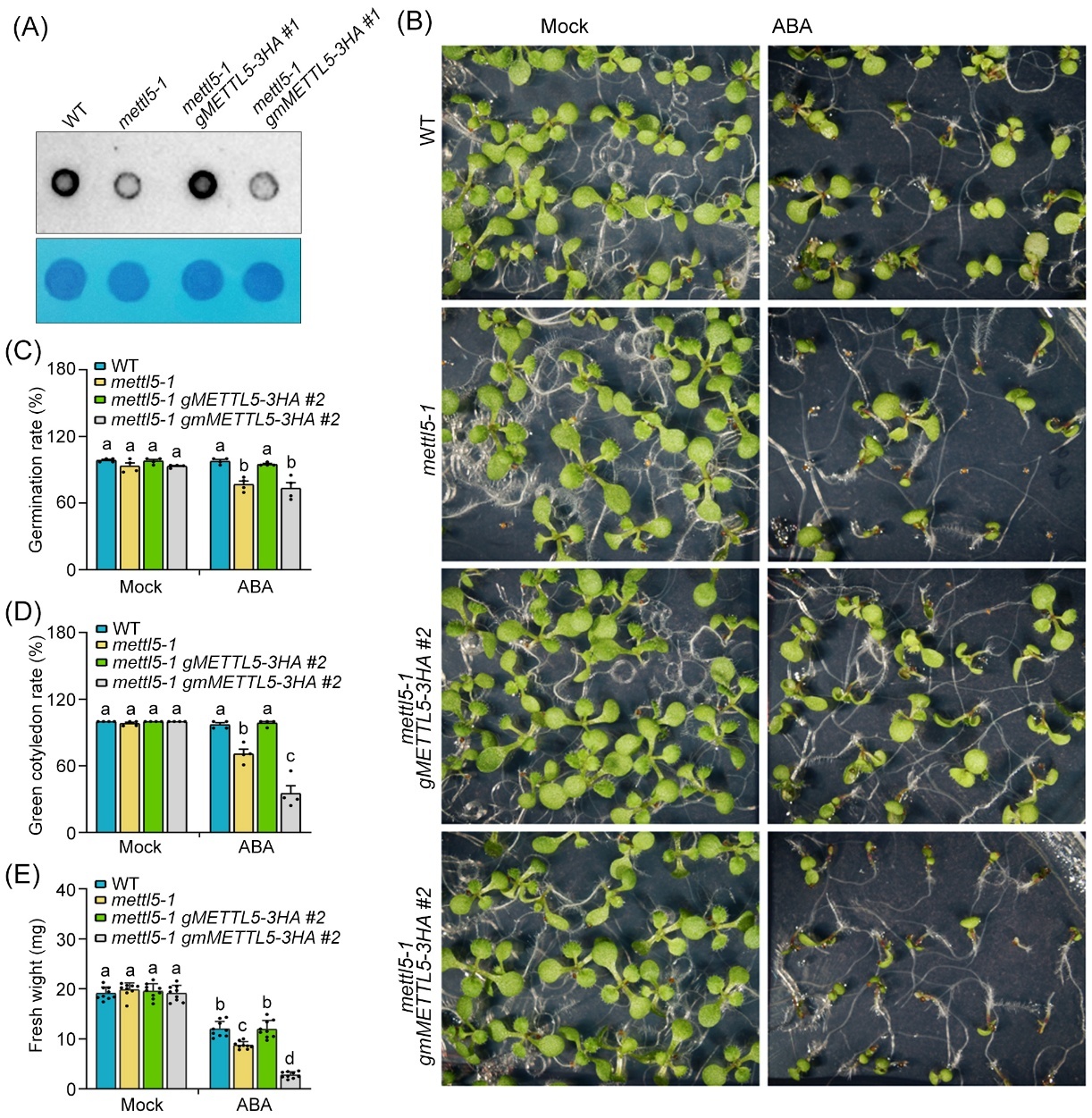


**Figure S6 *gMETTL5-3HA* but not *gmMETTL5-3HA* recuses the ABA hypersensitivity of *mettl5-1*.** (A) Dot blot analysis of total m^6^A levels in WT, *mettl5-1*, *mettl5-1 gMETTL5-3HA* *#1*, and *mettl5-1 gmMETTL5-3HA #1*. 18S rRNA was purified from 6-day-old seedlings. (B) ABA sensitivity analysis of additional independent lines of *mettl5-1 gMETTL5-3HA* and *mettl5-1 gmMETTL5-3HA*. Seeds were germinated on ½MS medium with or without 0.5 µM ABA, and images were taken 10 days after growth. (C−E) Analysis of germination rate (C), green cotyledon rate (D), and total fresh weight of 8 plants (E) of the additional independent lines of *mettl5-1* *gMETTL5-3HA* and *mettl5-1 gmMETTL5-3HA* transgenic plants with or without ABA treatment. Germination rate was calculated 3 days after growth, while green cotyledon rate and fresh weight were recorded after 10 days of growth. Different letters indicate statistically significant differences (*p* < 0.05, one-way ANOVA test).

**
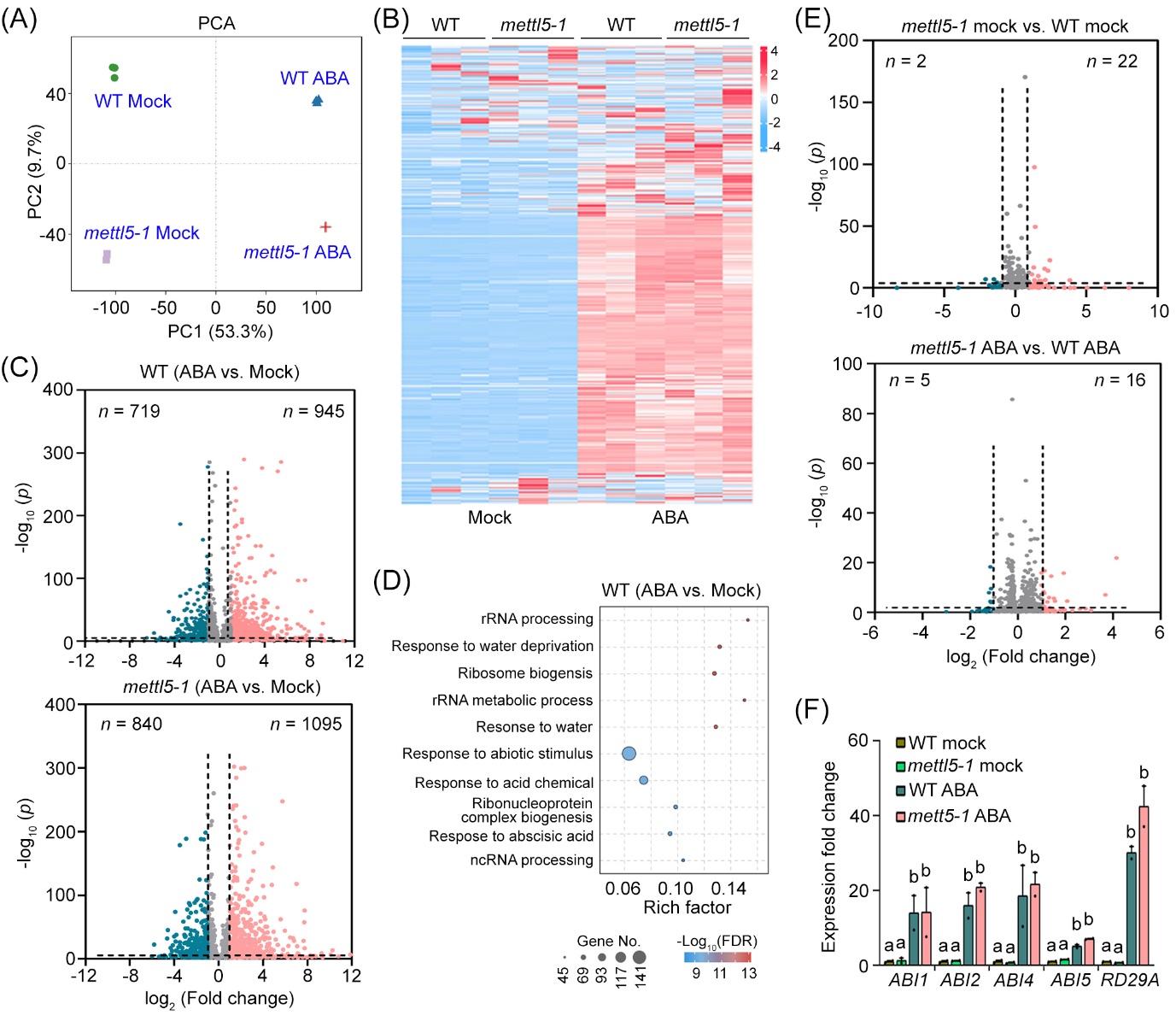
Figure S7** ***METTL5* has minor effects on global gene transcription.** (A) Principal component analysis (PCA) of transcriptomic data from WT and *mettl5-1* plants under mock and ABA treatments, highlighting distinct clusters based on genotype and treatment. (B) Heat map showing global gene expression among different samples. (C) Volcano plots showing DEGs in WT (top) and *mettl5-1* (bottom) plants under ABA treatment compared to mock conditions. The dashed lines indicate thresholds for statistical significance (-log_10_ (*p*) > 1.30103) and log_2_ (Fold change) > 1 or < -1. (D) GO enrichment analysis of DEGs under ABA treatment in WT and *mettl5-1*. (E) Volcano plots depicting differentially expressed genes (DEGs) in WT and *mettl5-1* under mock or ABA treatment. Black dotted lines represent the significance thresholds of -log_10_ (*p*) > 1.30103 and log_2_ (Fold change) > 1 or < -1. (F) qPCR analysis of the expression of ABA-responsive genes in WT and *mettl5-1* seedlings mock-treated or treated with 10 µM ABA for 5 h. Gene expression levels are normalized to *TUB2* expression and shown relative to levels in mock-treated WT plants set as 1.0. Different letters indicate statistically significant differences (*p* < 0.05, one-way ANOVA test).


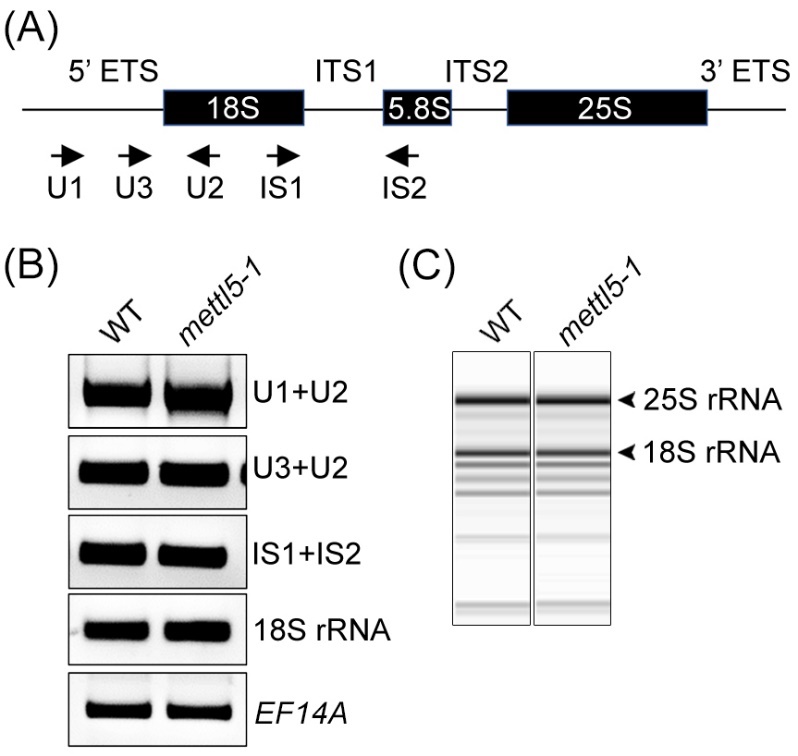


**Figure S8 METTL5-mediated m^6^A modification does not affect rRNA processing and abundance.** (A) Schematic representation of the rRNA precursor (pre-rRNA) structure, showing the 18S, 5.8S, and 25S rRNA coding regions flanked by external transcribed spacers (ETS) and internal transcribed spacers (ITS). Arrows indicate the locations of primers (U1, U2, U3, IS1, IS2) used for pre-rRNA analysis. (B) RT-PCR analysis of pre-rRNA processing intermediates in wild type (WT) and *mettl5-1* mutant plants. The amplified fragments correspond to U1+U2, U3+U2, IS1+IS2, and mature 18S rRNA, with *EF14A* used as a loading control. (C) Agilent Bioanalyzer analysis of total RNAs in WT and *mettl5-1* mutants.


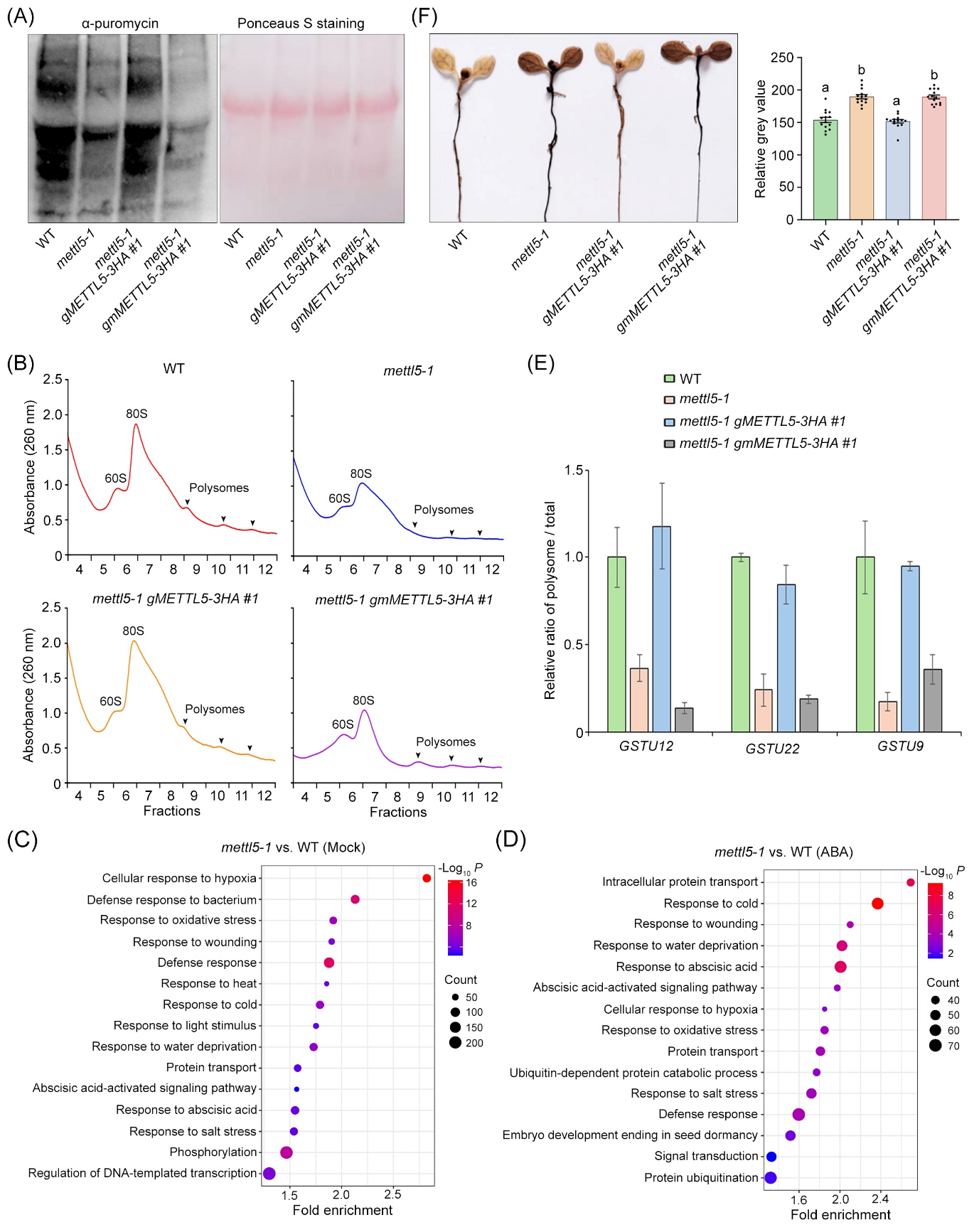
**Figure S9 *gMETTL5*, but not *gmMETTL5*, restores the defects in translational efficiency observed in *mettl5-1*.** (A) Detection of newly synthesized proteins using the SUnSET assay. Six-day-old seedlings of WT, *mettl5-1*, *mettl5-1 gMETTL5-3HA #1*, and *mettl5-1 gmMETTL5-3HA #1* were incubated with 50 μM puromycin for 1 hour. Total protein was separated on a 15% SDS-PAGE gel, and puromycin-labelled newly synthesized proteins were detected using anti-puromycin antibody. RbcL stained with Ponceau S served as a loading control. (B) Polysome profiling of WT, *mettl5-1*, *mettl5-1 gMETTL5-3HA #1*, *and mettl5-1 gmMETTL5-3HA #1* seedlings. (C and D) Gene Ontology (GO) analysis of differentially translated genes in WT and *mettl5-1* seedlings under mock (C) or ABA (D) treatment. (E) qPCR analysis of the translation efficiency of selected *GST* genes in WT, *mettl5-1*, *mettl5-1 gMETTL5-3HA #1*, and *mettl5-1 gmMETTL5-3HA #1* seedlings. (F) ROS accumulation visualized by DAB staining in WT, *mettl5-1*, *mettl5-1 gMETTL5-3HA #1*, and *mettl5-1 gmMETTL5-3HA #1* seedlings. The relative staining intensity of DAB quantified by Image J was shown on the left.


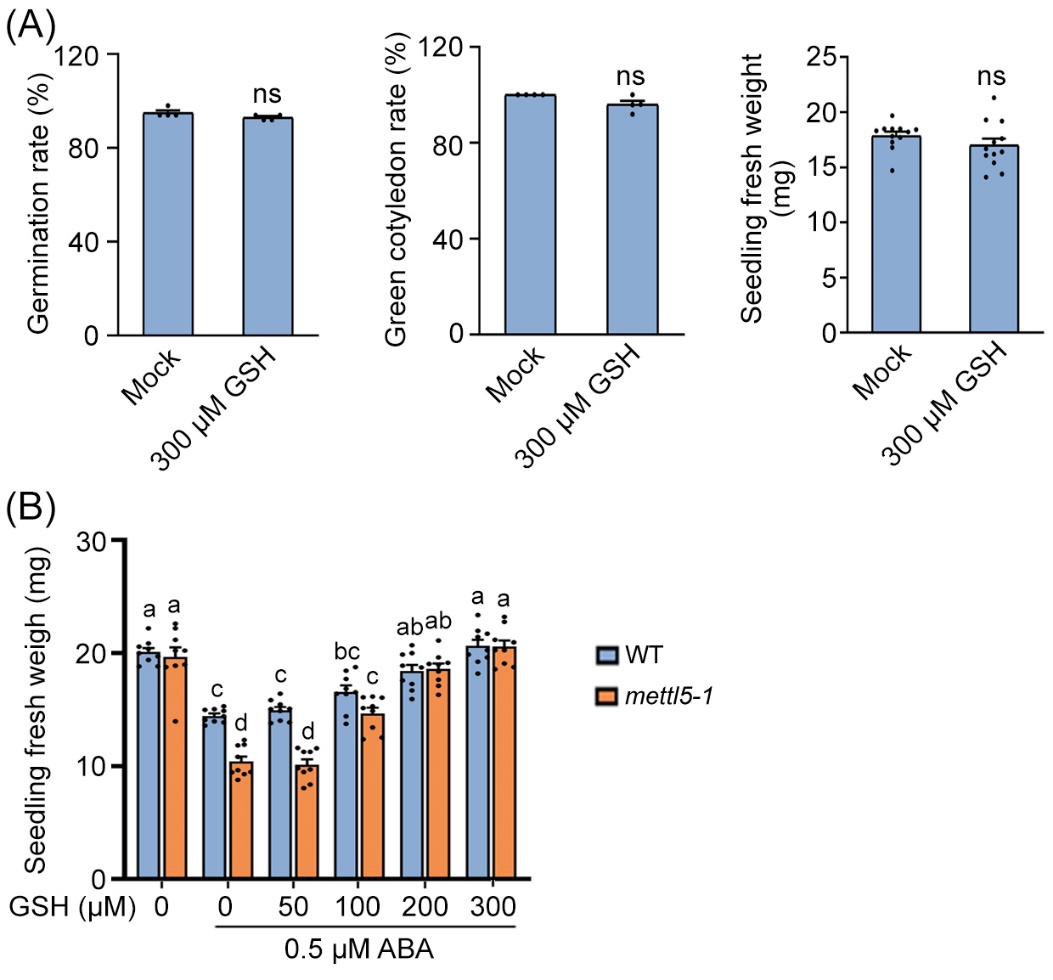


**Figure S10 Effect of GSH on plant growth.** (A) Exogenous addition of GSH does not affect seed germination or growth of *mettl5-1* mutants. Germination rate was calculated 2 days after growth, while green cotyledon rate and fresh weight were recorded after 10 days of growth. ns indicates no significant differences between mock and GSH treatments (ns, *p* > 0.05, two-tailed paired Student’s *t* test). (B) Analysis of total fresh weight of 8 plants of WT and *mettl5-1* in the presence of GSH under ABA treatment. Fresh weight was recorded after 10 days of growth. Different letters indicate statistically significant differences (*p* < 0.05, one-way ANOVA test).


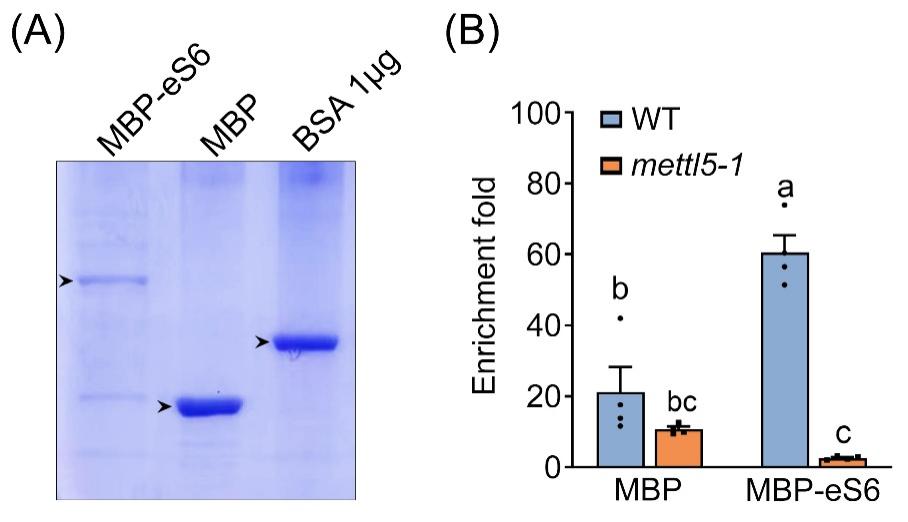


**Figure S11 The affinity between eS6 and 18S rRNA is decreased in *mettl5-1* mutants.** (A) Purified recombinant MBP-eS6 and MBP proteins. BSA, Bovine serum albumin. (B) *In vitro* RNA immunoprecipitation assay was conducted with MBP and MBP-eS6, followed by qPCR analysis using primers against 18S rRNA. Different letters indicate statistically significant differences (*p* < 0.05, one-way ANOVA test).


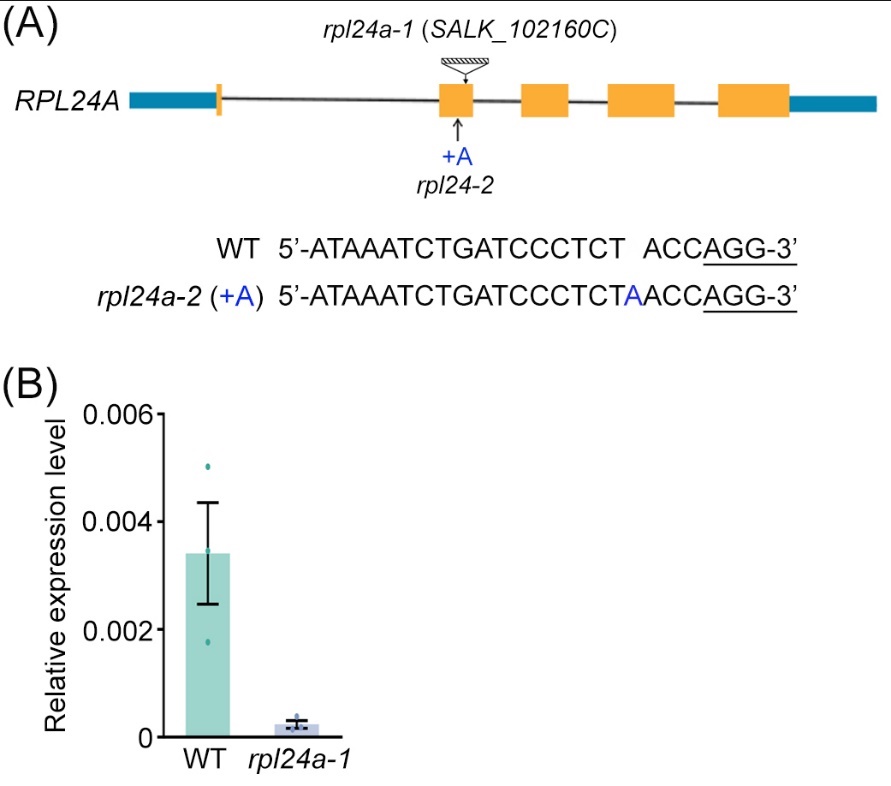


**Figure S12 Characterization of *RPL24A* mutants.** (A) Schematic representation of the *RPL24A* gene structure, indicating the locations of T-DNA insertion in *rpl24a-1* and mutation in *rpl24a-2*. Blue and yellow boxes represent UTRs and exons in the coding region, respectively, while black lines indicate introns. The lower panel shows alignment of wild-type (WT) and *rpl24a-2* sequences containing the CRISRP/Cas9 target sites with underlined protospacer adjacent motifs. *rpl24a-2* has a 1-bp insertion (A). (B) Relative expression levels of *RPL24A* in *rpl24a-1* mutant plants.


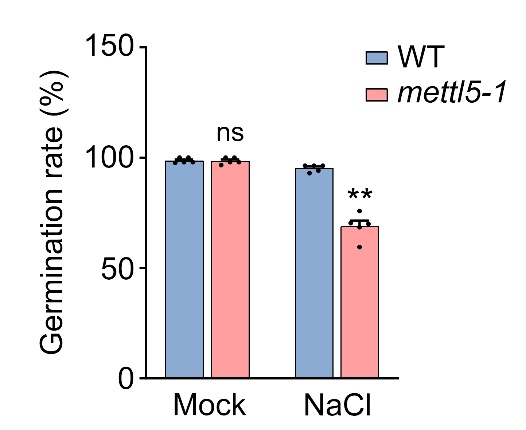


**Figure S13 *mettl5* mutants are hypersensitive to salt stress.** Germination rate was calculated 3 days after growth on ½ MS medium with or without 100 mM NaCl. Asterisks or ns indicate significant or no significant differences (two-tailed paired Student’s *t* test, ***p* < 0.01; ns, *p* > 0.05), respectively.


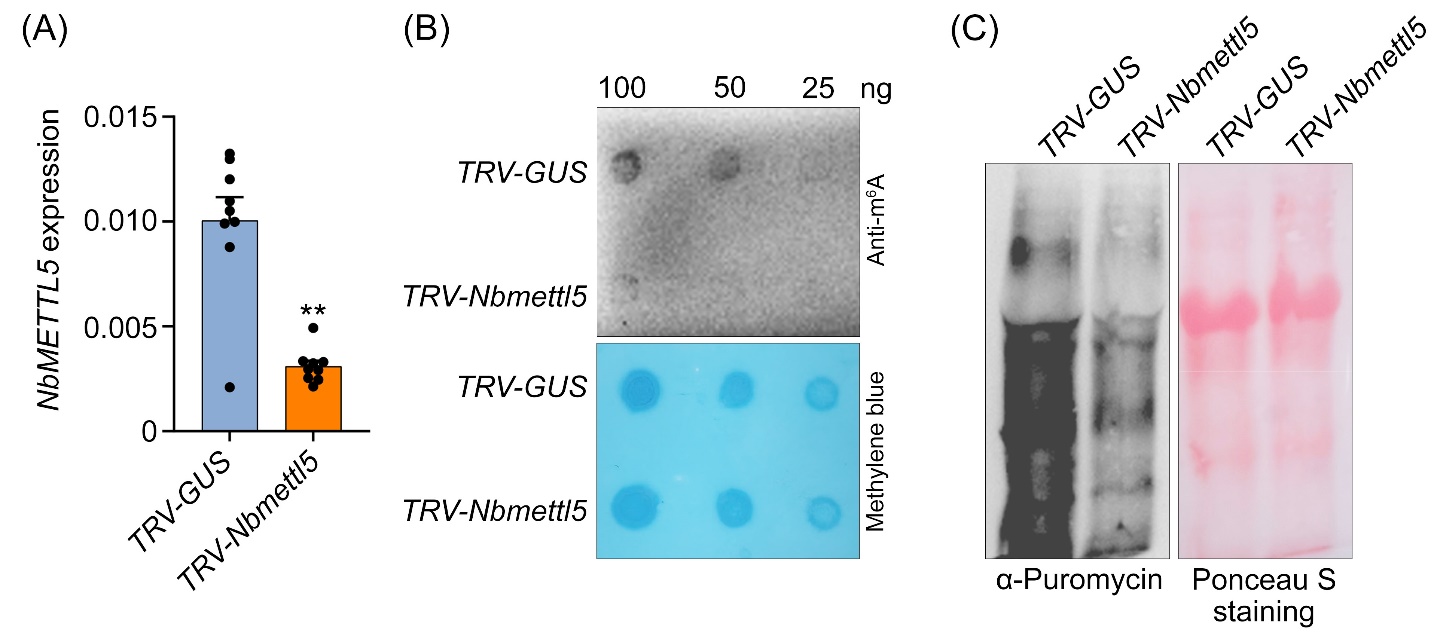
**Figure S14 Silencing of *NbMETTL5* reduces 18S rRNA m^6^A levels and global translation in *N. benthamiana*.** (A) *NbMETTL5* expression levels in *TRV-GUS* (control) and *TRV-Nbmettl5* plants measured by RT-qPCR. Asterisk indicates a significant reduction in *NbMETTL5* expression in *TRV-Nbmettl5* compared to *TRV-GUS* (***p* < 0.01, Student’s *t*-test). (B) Dot blot analysis of m^6^A levels in 18S rRNAs isolated from *TRV-GUS* and *TRV-Nbmettl5* plants. (C) Detection of newly synthesized proteins using the SUnSET assay. Puromycin-labelled newly synthesized proteins were detected using anti-puromycin antibody. RbcL stained with Ponceau S served as a loading control.


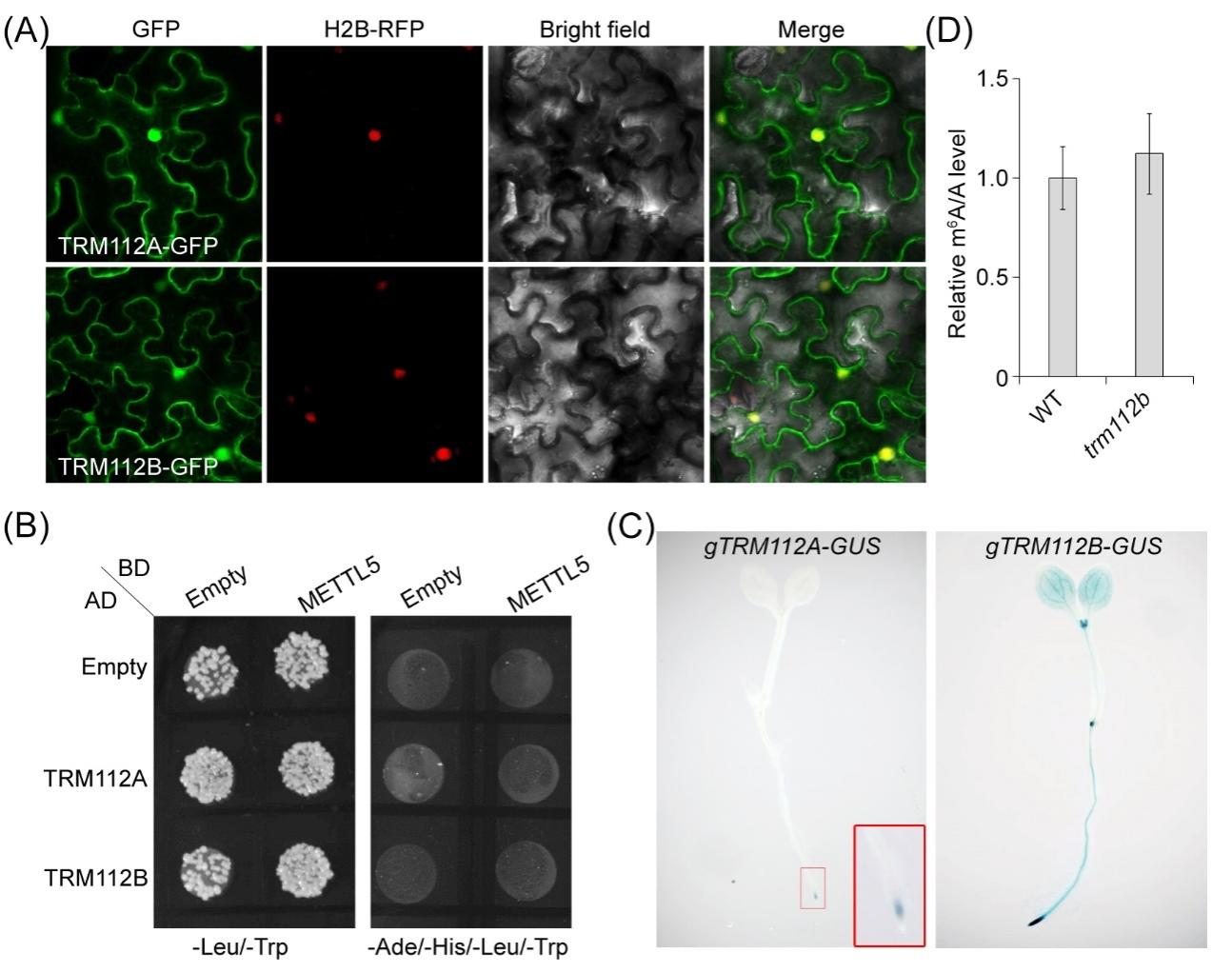
**Figure S15** **TRM112A/B does not interact with METTL5.** (A) Subcellular localization of TRM112A-GFP and TRM112B-GFP fusion proteins in *N. benthamiana* leaf epidermal cells. H2B-RFP, RFP fluorescence of the nuclear reporter (core histone 2B fused to red fluorescent protein). (B) Yeast two-hybrid assay showing no interaction between TRM112A or TRM112B and METTL5. (C) GUS staining of *gTRM112A-GUS* and *gTRM112B-GUS* transgenic plants. The inset shows an enlarged view of the root tip region. (D) LC-MS/MS quantification of m^6^A/A in 18S rRNA in *trm112b*. WT values were normalized to 1.0. Error bars, mean ± SE; *n* = 2 biological replicates.
